# The death burden of colorectal cancer attributable to modifiable risk factors, trend analysis from 1990 to 2019 and future predictions

**DOI:** 10.1002/cam4.7136

**Published:** 2024-03-28

**Authors:** Ning Zhu, Yan Zhang, Mi Mi, Yuwei Ding, Shanshan Weng, Jia Zheng, Yang Tian, Ying Yuan

**Affiliations:** ^1^ Key Laboratory of Cancer Prevention and Intervention, Ministry of Education, Department of Medical Oncology, Cancer Institute The Second Affiliated Hospital of Zhejiang University, School of Medicine Hangzhou Zhejiang China; ^2^ Department of Medical Geriatrics The Second Affiliated Hospital of Zhejiang University, School of Medicine Hangzhou Zhejiang China; ^3^ Department of Hepatobiliary and Pancreatic Surgery The Second Affiliated Hospital of Zhejiang University, School of Medicine Hangzhou Zhejiang China; ^4^ Zhejiang Provincial Clinical Research Center for Cancer Hangzhou Zhejiang China; ^5^ Cancer Center, Zhejiang University Hangzhou Zhejiang China

**Keywords:** age standardized death rate, average annual percentage change, colorectal cancer, Global Burden of Disease Study, population attributable fraction, risk factor

## Abstract

**Background:**

The death burden attributable to modifiable risk factors is key to colorectal cancer (CRC) prevention. This study aimed to assess the prevalence and regional distribution of attributable CRC death burden worldwide from 1990 to 2019.

**Methods:**

We extracted data from the Global Burden of Disease Study in 2019 and assessed the mortality, age‐standardized death rate (ASDR), population attributable fractions, and time trend in CRC attributable to risk factors by geography, socio‐demographic index (SDI) quintile, age, and sex.

**Results:**

Over the past 30 years, from high to low SDI region, the number of deaths increased by 46.56%, 103.55%, 249.64%, 231.89%, 163.11%, and the average annual percentage change (AAPC) for ASDR were −1.06%, −0.01%, 1.32%, 1.19%, and 0.65%, respectively. ASDR in males was 1.88 times than in females in 2019; ASDR in males showed an increasing trend (AAPC 0.07%), whereas ASDR in females showed a decreasing trend (AAPC −0.69%) compared to figures in 1990. In 2019, from high to low SDI region, the 15–49 age group accounted for 3%, 6%, 10%, 11%, and 15% of the total population; dietary and metabolic factors contributed 43.4% and 20.8% to CRC‐attributable death worldwide. From high to low SDI region, ASDRs caused by dietary and metabolic factors increased by −23.4%, −5.5%, 25.8%, 29.1%, 13.5%, and 1.4%, 33.3%, 100.8%, 128.4%, 77.7% respectively, compared to 1990.

**Conclusions:**

The attributable CRC death burden gradually shifted from higher SDI to lower SDI regions. The limitation in males was more significant, and the gap is expected to be further expanded. In lower SDI regions, the death burden tended to affect younger people. The leading cause of CRC‐attributable deaths was the inadequate control of dietary and metabolic risk factors.

## 1 INTRODUCTION

Colorectal cancer (CRC) is one of the world's most common and high‐burden cancers.^1^ According to the 2020 global cancer statistics, new cases of CRC had doubled over the past 30 years to an estimated 1.93 million new cases and resulted in 0.92 million deaths worldwide, and this burden continues to increase.^2^, ^3^

Exposure to risk factors plays a pivotal role in the biology and health burden of CRC. Studies have shown that only 25%–30% of colorectal cancers are associated with non‐modifiable risk factors such as genetic factors, and the vast majority of sporadic colorectal cancers are associated with modifiable risk factors.^4^, ^5^, ^6^, ^7^ Currently, it is believed that the occurrence and development of CRC are the most closely related to modifiable risk factors,^8^ such as smoking, alcohol consumption, obesity, hyperglycemia, and unreasonable diet structure.^9^, ^10^, ^11^ Approximately 50%–60% of CRC cases in the United States were reported as attributable to these modifiable factors.^12^ In recent years, the global dietary structure and lifestyle have changed due to the rapid economic development and social progress, exerting a broad and far‐reaching impact on the CRC burden.^13^

While a portion of CRC cases cannot be prevented, at the government level, a range of efforts can be undertaken to reduce the population's exposure to known cancer risk factors and environmental agents.^14^ Primary cancer prevention or the prevention of cancer development are particularly cost‐effective strategies, while combined with secondary prevention initiatives such as stool testing, colorectal screening, etc., can have profound implications for the prevention and control of CRC.^15^, ^16^ Multiple studies have confirmed that colorectal cancer can be prevented by improving modifiable risk factors, and death can be prevented by early screening interventions to detect polyps.^17^, ^18^, ^19^ Therefore, understanding the relative contribution of modifiable risk factors to the burden of colorectal cancer and their trends over time, assessing the latest trends at global, regional, and national levels, as well as the associated risk factors in countries at different levels of development, can help track progress, map resource needs, and be essential to help develop and implement effective policies to prevent and respond to the growing burden of colorectal cancer locally and globally.^20^

Although data on CRC incidence, mortality, and risk factors were updated in 2019 by the Global Burden of Disease Study Group (hereinafter referred to as GBD 2019), the analysis of risk factors provides only a brief description of the percentage of risk factors contributing to colorectal cancer disability‐adjusted life years of all ages globally and regionally in 2019.^20^ The CRC burden attributable to modifiable risk factors and its epidemic trends and patterns have not been explored in depth, and no relevant analysis has been conducted to date. Due to the increasing CRC burden, a comprehensive and up‐to‐date analysis of the epidemiological characteristics and geographical distribution of the attributable CRC death burden is urgently needed, which can provide a breakthrough in colorectal cancer prevention and control.

To address the above shortfall, this study used GBD2019 data to analyze the trends and prevalence characteristics of CRC death burden attributable to risk factors in different SDI regions, 21 GBD regions, and 204 countries and regions from 1990 to 2019, and made predictions of future trends, providing a reliable theoretical guarantee for the development of local tailored primary prevention policies.

## 2 MATERIALS AND METHODS

### 2.1 Data source

Data on the CRC death burden attributable to risk factors from 1990 to 2019 were obtained from the GBD tool (https://ghdx.healthdata.org/gbd‐results‐tool). We collected data on CRC‐attributable deaths, the age‐standardized death rate (ASDR), population attributable fraction (PAF), and their uncertainty intervals (UI) for 5 socio‐demographic index (SDI) regions, 21 GBD regions, and 204 countries and territories, and analyzed the epidemiological characteristics and trends by different regions, age, sex, and risk factors. The age‐standardized rate (ASR) expresses the number of people per 100,000 population. The time trends in burden over the study period were assessed by the rate of change in deaths and PAFs and the average annual percentage change (AAPC) of ASDR from 1990 to 2019.

The SDI measures the development level of a country comprehensively by per capita income level, average education level, and total fertility rate, ranging from 0 to 1.^21^, ^22^ According to the SDI value in 2019, countries and regions were divided into five categories (high SDI >0.81, high middle SDI 0.70–0.81, middle SDI 0.61–0.69, low middle SDI 0.46–0.60, and low SDI <0.46).

### 2.2 Modifiable risk factors

This study uses the comparative risk assessment framework of GBD 2019 to assess the disease burden attributable to the 87 health risk factors.^23^, ^24^ The attributable risk in CRC was divided into five categories: smoking, low physical activity, alcohol, dietary risks, and metabolic factors. Among them, dietary factors were classified into diet high in red meat, diet high in processed meat, diet low in whole grains, diet low in milk, diet low in calcium and diet low in fiber, and metabolic factors were divided into high body‐mass index, high fasting plasma glucose, and totaling 11 items. Data on risk factors were extracted from 46,000 empirical data points obtained from cohort studies and randomized controlled trials.

### 2.3 Population attributable fraction

For each risk factor *j*, we computed the population attributable fraction (PAF) by age‐sex‐location‐year using the following general formula for a continuous risk:

$${\mathrm{PAF}}_{\text{joasgt}}=\frac{\int_{x=l}^{u}RR_{\text{joasg}}\left(x\right)P_{\text{joasg}}\left(x\right)\mathrm{dx}-RR_{\text{joasg}}\left({\text{TMREL}}_{\mathrm{jas}}\right)}{\int_{x=l}^{u}RR_{\text{joasg}}\left(x\right)P_{\text{joasg}}\left(x\right)\mathrm{dx}}$$

where *PAF*_*joasgt*_ represents the *PAF* for cause *o*, age group *a*, sex *s*, location *g*, and year *t*; RR_joasg_ (*x*) represents the relative risk as a function of exposure level *x* for risk factor *j*, for cause *o* controlled for confounding, age group *a*, sex *s*, and location *g* with the lowest level of observed exposure as *l* and the highest as *u*; *P*_*jasgt*_ (*x*) represents the distribution of exposure at *x* for age group *a*, sex *s*, location *g*, and year *t*; and TMREL_jas_ represents the TMREL for risk factor *j*, age group *a*, and sex *s*. When the risk exposure is dichotomous or polytomous, this formula simplifies to the discrete form of the equation.^24^

All PAFs data were taken from GBD 2019, and we have analyzed the population attributable fraction of different risk factors and their changes.

### 2.4 Prediction in death rate

In order to predict mortality by sex from 2019 to 2030, the recent trends were fitted with a log‐linear age‐period‐cohort model that eliminates exponential growth and limits linear trend predictions. The model, implemented in R language via the NORDPRED package, has been shown to perform well in predicting current trends in future cancer mortality.^25^, ^26^ We expected the CRC death rate to be attributable to different classifications of risk factors by 2030.

### 2.5 Ethics statement

The data used in this study are publicly available, and patient information was based on the summarized data rather than the individual level. This study was in line with one of the items exempted from review by the Ethics Committee, that is, “Previous data research: collection of existing data, records, discarded pathological specimens or examination specimens, and these resources are public resources, or the information saved by the researcher will not identify the subject, nor will it directly or indirectly identify the subject.” Therefore, the Human Research Ethics Committee of the Second Affiliated Hospital of Zhejiang University School of Medicine decided that the study did not require ethical review.

### 2.6 Statistical analysis

Regression analysis was performed to calculate the average annual percentage change (AAPC) and 95% confidence interval (CI) for ASDR using Joinpoint 4.2.0.1 software. The correlation between ASDR, PAF and SDI in 21 GBD regions, maps of ASDR and AAPC, PAFs, the change rates in 204 countries and territories, and the prediction of ASDRs attributable to risk factors by SDIs were performed in R (version 4.1.2). Statistical tests were two–sided, and *p* < 0.05 was considered significant.

## 3 RESULTS

### 3.1 Attributable CRC death burden in SDI regions, 21GBD regions and 204 countries and territories

**FIGURE 1:**
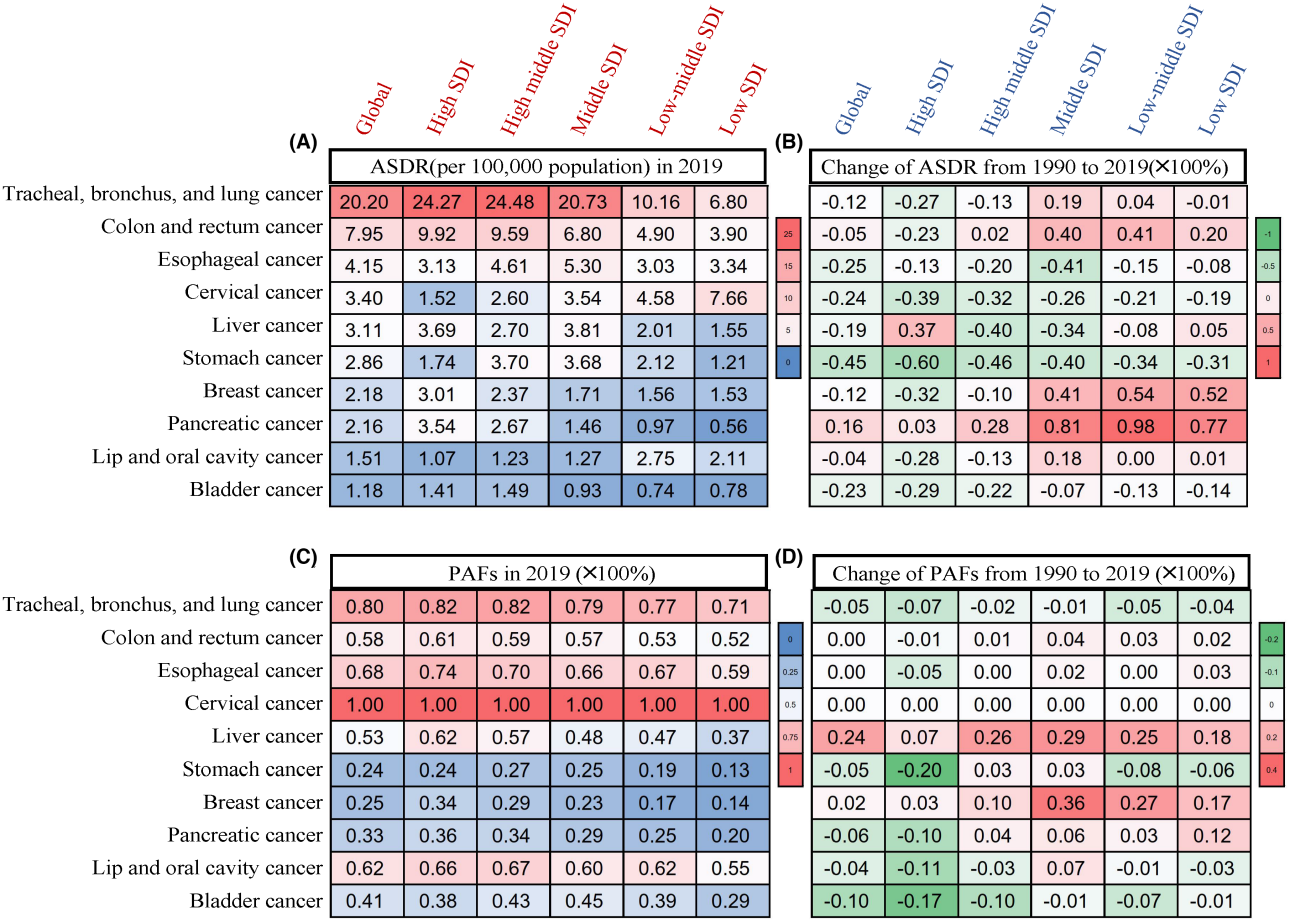
ASDR and PAFs of the top 10 cancers in ASDR attributable to all risk factors in 2019 and their variations from 1990 to 2019 by SDIs. (A) ASDR of the top 10 cancers in ASDR attributable to all risk factors by SDIs in 2019. (B) Changes in the ASDR of top 10 cancers in ASDR attributable to all risk factors by SDIs from 1990 to 2019. (C) PAFs of top 10 cancers in ASDR attributable to all risk factors by SDIs in 2019. (D) Changes in the PAF of top 10 cancers in ASDR attributable to all risk factors by SDIs from 1990 to 2019.

In 2019, among ASDR attributable to risk factor burden in cancers, CRC ranked second worldwide, and the trend in ASDR and PAFs showed significant regional heterogeneity (Figure 1). In global terms, there were 301,767 (95% UI, 266,647–337,026) attributable CRC deaths in 1990 and 631,750 (95% UI, 550,544–731,603) in 2019. The ASDR attributable to risk factors gradually decreased from 8.34 (95% UI, 7.36–9.36) per 100,000 population in 1990 to 7.95 (95% UI, 6.89–9.09) in 2019, and the AAPC was −0.21% (95% UI, −0.24% to 0.19%) compared to 1990. The PAFs were stable for 58% (Table 1; Figure 1).

**TABLE 1:** The death cases, ASDR and PAFs of CRC attributable to all risk factors in 1990 and 2019 and their variations from 1990 to 2019.

	1990			2019			1990–2019		
	Death number	ASDR	PAF	Death number	ASDR	PAF	Change of number	AAPC	Change of PAF
	(95% UI, *n*)	(95% UI)^a^	(×100%)	(95% UI, *n*)	(95% UI)^a^	(×100%)	(×100%)	(95% UI, %)	(×100%)
Global	301766.64	8.34	0.58	631,750.14	7.95	0.58	1.09	−0.21	0
	(266647.73 ~ 337025.68)	(7.36–9.36)	(0.52–0.65)	(550,544.36–721,603.33)	(6.89–9.09)	(0.51–0.65)	(0.95–1.23)	(−0.24–−0.19)	(−0.03–0.02)
Sex									
Male	166337.92	10.46	0.65	382892.71	10.7	0.64	1.3	0.07	0
	(148,526.52–184,024.76)	(9.32–11.65)	(0.58–0.71)	(332,744.71–435,104.52)	(9.32–12.17)	(0.58–0.71)	(1.09–1.5)	(0.04–0.1)	(−0.02–0.02)
Female	135428.72	6.73	0.52	248857.43	5.68	0.51	0.84	−0.69	−0.03
	(115,469.01–154,848.52)	(5.71–7.71)	(0.45–0.59)	(204,200.76–295,747.37)	(4.66–6.75)	(0.42–0.59)	(0.69–0.98)	(−0.72–−0.66)	(−0.06–0.01)
SDI									
High SDI	135363.66	12.87	0.61	198392.64	9.92	0.61	0.47	−1.06	−0.01
	(118,804.9–152,008.62)	(11.32–14.47)	(0.54–0.68)	(169,767.93–229,313.53)	(8.59–11.37)	(0.53–0.68)	(0.39–0.54)	(−1.09–−1.04)	(−0.03–0.02)
High‐middle SDI	95119.42	9.37	0.58	193613.09	9.59	0.59	1.04	−0.01	0.01
	(84,080.67–106,594.5)	(8.27–10.5)	(0.52–0.65)	(167,358.58–221,236.33)	(8.28–10.96)	(0.52–0.67)	(0.88–1.19)	(−0.05–0.03)	(−0.01–0.04)
Middle SDI	45,494.48	4.86	0.55	159,065	6.8	0.57	2.5	1.32	0.04
	(39,555.33–51,641.84)	(4.21–5.53)	(0.48–0.61)	(135,040.54–185,254.68)	(5.79–7.91)	(0.5–0.64)	(2.05–2.95)	(1.27–1.37)	(0–0.08)
Low‐middle SDI	18746.91	3.48	0.52	62219.22	4.9	0.53	2.32	1.19	0.03
	(15,979.84–22,158.61)	(2.96–4.09)	(0.46–0.58)	(52,606.58–73,150.37)	(4.13–5.75)	(0.47–0.61)	(1.77–2.8)	(1.18–1.21)	(−0.02–0.07)
Low SDI	6878.63	3.26	0.51	18098.31	3.9	0.52	1.63	0.65	0.02
	(5563.73–8381.64)	(2.65–3.95)	(0.45–0.57)	(15,186.59–21,183.3)	(3.27–4.55)	(0.46–0.59)	(1.17–2.15)	(0.63–0.66)	(−0.01–0.06)
GBD Region									
High‐income Asia Pacific	19,667.83	10.19	0.57	42837.38	8.62	0.56	1.18	−0.7	−0.03
	(17,247.57–22,131.31)	(8.88–11.51)	(0.51–0.64)	(35,141.37–50,740.27)	(7.18–10.02)	(0.48–0.63)	(0.95–1.33)	(−0.73–−0.67)	(−0.06–0.01)
Central Asia	2799.19	5.99	0.55	4226.31	6.3	0.57	0.51	0.39	0.04
	(2425.83–3131.75)	(5.2–6.72)	(0.48–0.61)	(3591.09–4938.03)	(5.33–7.38)	(0.49–0.64)	(0.37–0.68)	(0.33–0.45)	(0–0.08)
East Asia	45,243.62	5.65	0.54	157516.41	7.99	0.57	2.48	1.51	0.05
	(37,985.9–53,020.38)	(4.75–6.62)	(0.48–0.61)	(127,148.58–189,347.21)	(6.48–9.55)	(0.5–0.64)	(1.87–3.25)	(1.42–1.6)	(0–0.11)
Southeast Asia	13,160.64	5.57	0.56	47,508.26	8.39	0.58	2.61	1.35	0.04
	(11,260.3–15,127.73)	(4.78–6.38)	(0.49–0.62)	(37,638.4–58,150.98)	(6.61–10.27)	(0.51–0.65)	(1.97–3.24)	(1.33–1.37)	(0–0.08)
South Asia	13,422.28	2.75	0.49	47619.26	3.73	0.5	2.55	0.92	0.02
	(11,171.77–16,216.72)	(2.29–3.32)	(0.43–0.55)	(38,348.73–57,895.12)	(3.01–4.52)	(0.43–0.58)	(1.8–3.26)	(0.88–0.96)	(−0.03–0.08)
Australasia	3567.99	15.4	0.63	5296.93	10.29	0.63	0.48	−1.7	0
	(3176.69–3969.46)	(13.67–17.13)	(0.56–0.69)	(4523.3–6031.88)	(8.87–11.7)	(0.56–0.7)	(0.38–0.59)	(−1.75–−1.66)	(−0.04–0.03)
Oceania	116.29	4.45	0.56	319.61	5.2	0.58	1.75	0.52	0.03
	(88.45–146.68)	(3.42–5.56)	(0.49–0.64)	(244.4–415.08)	(4.05–6.62)	(0.5–0.66)	(1.22–2.41)	(0.5–0.54)	(−0.01–0.07)
North Africa and Middle East	7214.85	4.59	0.55	22,962.98	5.81	0.59	2.18	1.02	0.06
	(5780.41–8862.37)	(3.68–5.64)	(0.48–0.62)	(18,918.59–27,799.16)	(4.8–7)	(0.51–0.66)	(1.54–3.06)	(0.95–1.08)	(0.02–0.11)
Central Sub‐Saharan Africa	795.2	4.04	0.52	1938.36	4.14	0.55	1.44	0.01	0.04
	(619.54–1032.33)	(3.11–5.21)	(0.46–0.59)	(1438.26–2580.03)	(3.05–5.63)	(0.48–0.61)	(0.69–2.36)	(−0.08–0.1)	(0.01–0.08)
Eastern Sub‐Saharan Africa	2431.32	3.54	0.5	6382.71	4.3	0.5	1.63	0.7	0.01
	(1968.77–2978.07)	(2.86–4.33)	(0.43–0.55)	(5171–7798.37)	(3.53–5.26)	(0.44–0.56)	(1.11–2.25)	(0.68–0.73)	(−0.02–0.05)
Southern Sub‐Saharan Africa	1525.95	6.07	0.59	3543.41	6.9	0.6	1.32	0.46	0.01
	(1277.33–1891.05)	(5.02–7.59)	(0.53–0.66)	(3002.3–4161.82)	(5.85–8.1)	(0.53–0.67)	(1.04–1.71)	(0.38–0.54)	(−0.03–0.04)
Western Sub‐Saharan Africa	2698.35	3.47	0.52	7238.03	4.48	0.53	1.68	1.06	0.01
	(2125.03–3377.5)	(2.74–4.33)	(0.45–0.58)	(5738.52–8814.35)	(3.62–5.42)	(0.46–0.59)	(1.18–2.36)	(1.03–1.09)	(−0.03–0.05)
Central Europe	18,510.43	12.82	0.6	31995.71	14.61	0.62	0.73	0.47	0.03
	(16,346.4–20,728.22)	(11.26–14.38)	(0.53–0.67)	(26,813.71–38,184.62)	(12.24–17.38)	(0.54–0.7)	(0.53–0.94)	(0.43–0.5)	(0.01–0.06)
Eastern Europe	28,138.71	10.11	0.56	36,312.52	10.45	0.57	0.29	−0.32	0.01
	(24,741.6–31,341.94)	(8.87–11.27)	(0.49–0.63)	(30,930.67–41,822.25)	(8.88–12.03)	(0.5–0.64)	(0.16–0.43)	(−0.4–−0.24)	(−0.04–0.05)
Western Europe	82,522.68	13.99	0.63	109,159	11.02	0.63	0.32	−1.06	0
	(72,912.72–92,208.24)	(12.38–15.61)	(0.56–0.7)	(93,306.41–125,522.8)	(9.51–12.56)	(0.55–0.71)	(0.25–0.39)	(−1.11–−1)	(−0.02–0.03)
High‐income North America	43,306.73	12.08	0.6	58,889.26	9.22	0.62	0.36	−1.06	0.02
	(37,363.62–49,328.96)	(10.44–13.73)	(0.52–0.68)	(50,189.62–68,051.76)	(7.92–10.6)	(0.53–0.7)	(0.3–0.43)	(−1.09–−1.03)	(−0.01–0.06)
Caribbean	1895.07	7.58	0.58	4704.33	9.1	0.59	1.48	0.67	0.02
	(1633.85–2158.62)	(6.52–8.67)	(0.5–0.66)	(3790.77–5799.65)	(7.34–11.22)	(0.5–0.68)	(1.15–1.84)	(0.65–0.69)	(−0.01–0.05)
Andean Latin America	780.91	4.08	0.51	2957.93	5.43	0.53	2.79	1.23	0.02
	(645.97–911.1)	(3.38–4.8)	(0.44–0.58)	(2275.69–3756.76)	(4.18–6.89)	(0.45–0.6)	(2.08–3.65)	(1.18–1.28)	(−0.02–0.07)
Central Latin America	3147.57	4.06	0.55	12245.79	5.3	0.54	2.89	0.89	−0.01
	(2693.67–3642.28)	(3.48–4.74)	(0.47–0.63)	(9722.65–15204.2)	(4.23–6.59)	(0.46–0.64)	(2.37–3.5)	(0.88–0.91)	(−0.04–0.02)
Tropical Latin America	5187.3	6.22	0.61	16,813.28	7.08	0.61	2.24	0.53	−0.01
	(4578.14–5842.58)	(5.47–7.03)	(0.54–0.68)	(14,445.91–19,176.08)	(6.08–8.1)	(0.53–0.68)	(2.03–2.44)	(0.47–0.59)	(−0.04–0.03)
Southern Latin America	5633.73	12.64	0.64	11282.67	13.35	0.63	1	0.17	−0.01
	(5027.94–6205.48)	(11.27–13.94)	(0.57–0.7)	(9856.57–12,827.57)	(11.67–15.16)	(0.55–0.7)	(0.88–1.14)	(0.14–0.2)	(−0.05–0.02)

Abbreviations: AAPC, average annual percentage change; ASDR, age‐standardized death rate; GBD, global disease burden; PAF, population attributable fraction; SDI, socio‐demographic index; UI, uncertainty interval.

^a^ Per 100,000 population.

Notably, the prevalence patterns of attributable CRC death burden changed in different SDI regions. Although the higher SDI (high, high‐middle SDI) regions still had the highest ASDR by 2019, its growth rate showed a negative increase (Figure 1B; Table 1). The burden gap gradually narrowed due to the more obviously increasing growth rate in the lower SDI (middle, low‐middle, and low SDI) regions (from high to low SDI, AAPC of ASDR = −1.06%, −0.01%, 1.32%, 1.19%, 0.65%, the change of PAFs = −0.53%, 1.37%, 4.18%, 2.67%, 2.01%) (Figure 1D; Table 1).

**FIGURE 2:**
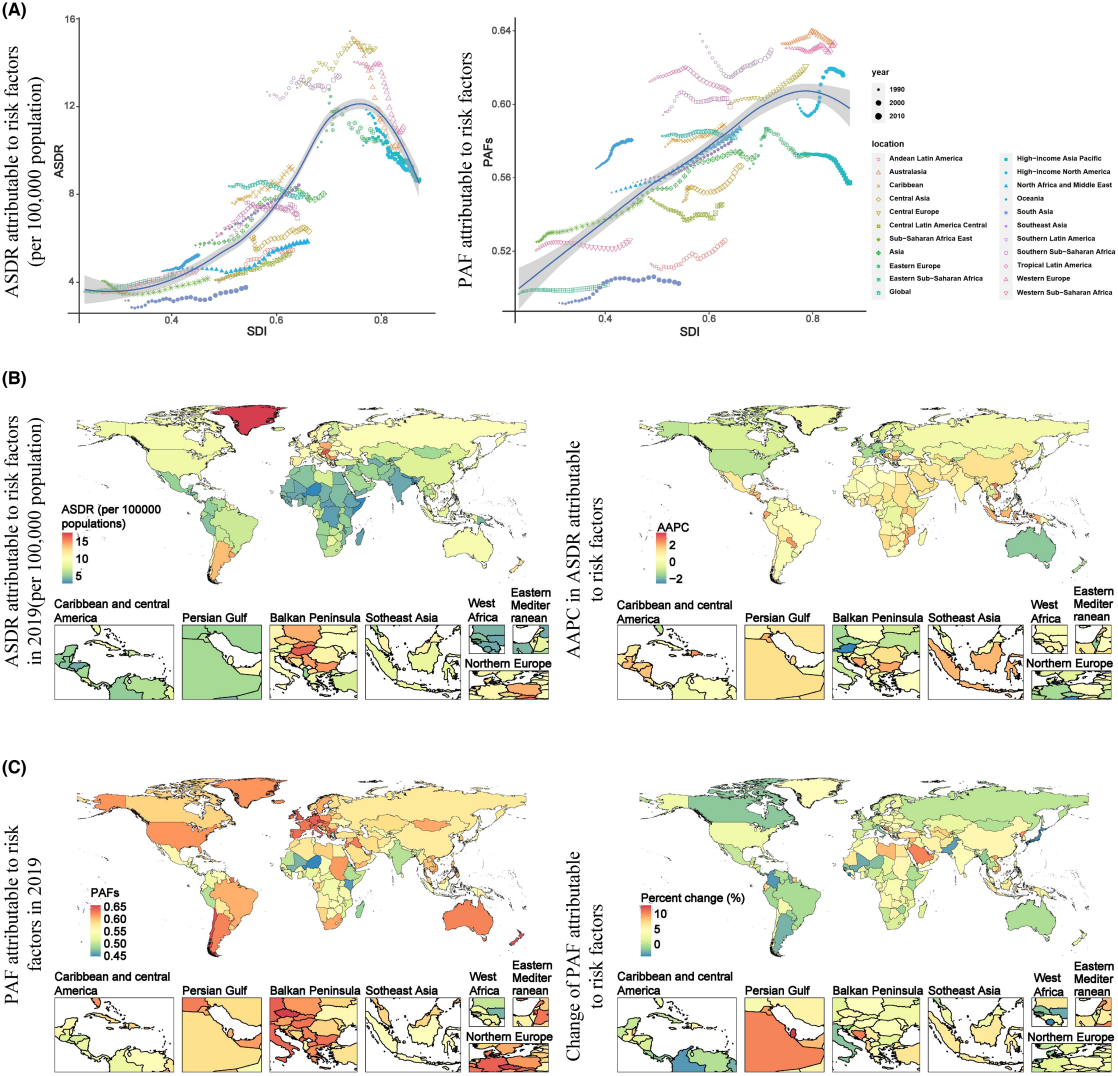
ASDR and PAFs attributable to all risk factors in CRC from 1990 to 2019. (A) ASDR and PAFs attributable to all risk factors in CRC by GBD regions from 1990 to 2019. (B) ASDR in 2019 and AAPC of ASDR from 1990 to 2019 in CRC attributable to all risk factors by countries and territories. (C) PAFs in 2019 and change in PAFs from 1990 to 2019 in CRC attributable to all risk factors by countries and territories.

Among the 21 GBD regions, Australasia (AAPC of ASDR = −1.7%), high‐income North America (AAPC of ASDR = −1.06%), Western Europe (AAPC of ASDR = −1.06%), high‐income Asia Pacific (AAPC of ASDR = −0.7%), and Eastern Europe (AAPC of ASDR = −0.32%) with higher SDI values showed a downward trend in attributable ASDR in CRC. In contrast, East Asia, Southeast Asia, North Africa and Middle East, Andean Latin America, Western Sub‐Saharan Africa, which are regions with relatively low SDI values, showed a clear upward trend in ASDR. East Asia presented the most obvious upward trend (AAPC of ASDR = 1.51%) (Table 1; Figure 2A).

Among 204 countries and territories, Greenland, Hungary, and Brunei Darussalam had the highest ASDR (19.2 (95% UI, 15.2–23.9)), 18.1 (95% UI, 14.5–22.5, 17.0 (95% UI, 13.0–21.4) per 100,000 population) in 2019, while the highest increase of ASDR occurred in Equatorial Guinea, Vietnam and Cabo Verde (AAPC = 3.69%, 2.95%, 2.66%) from 1990 to 2019. Czechia, New Zealand and Chile exhibited the highest PFAs (65.8% (95% UI, 57.2%–74.5%), 65.2% (95% UI, 58.4%–71.9%), 65.1% (95% UI, 57.2%–72.7%) in 2019), while the highest increase of PAFs occurred in Qatar, Bosnia and Herzegovina, and Saudi Arabia (change of PAFs = 13.2%, 10.9%, 10.7%) (Figure 2B,C; Table S1).

In conclusion, the attributable CRC death burden showed significant regional heterogeneity, with a downward trend in higher SDI regions and a significant upward trend in lower SDI regions.

### 3.2 Gender differences and trends in attributable CRC death burden

**FIGURE 3:**
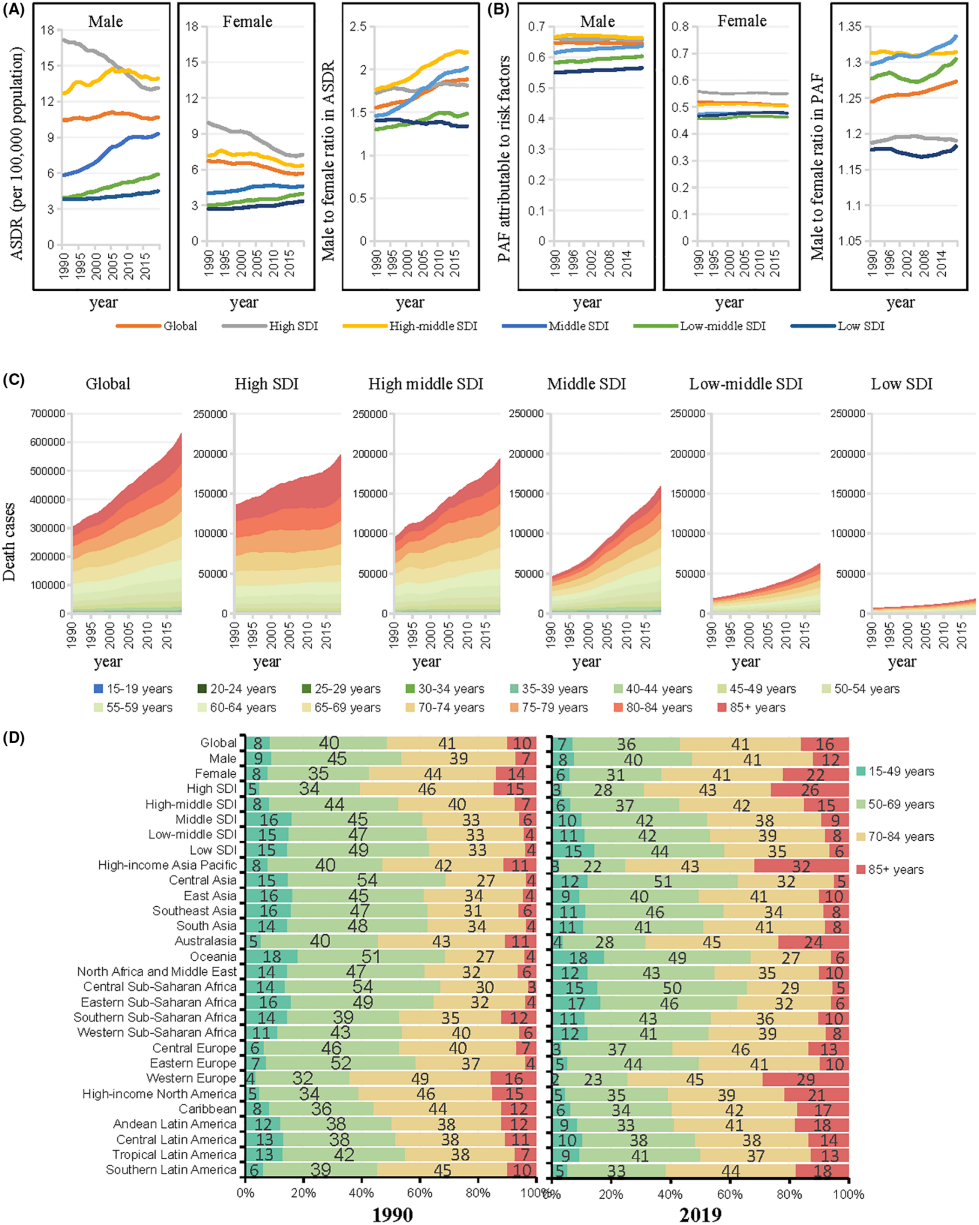
Age and sex differences and trends in CRC deaths attributable to all risk factors by different SDIs and GBD regions. (A) Trends in ASDR and PAFs attributable to all risk factors in CRC by SDIs in males, females and M/F ratio from 1990 to 2019. (B) Percentage of change to CRC deaths attributable to all risk factors by SDIs and GBD regions between 1990 and 2019 in four age groups. (C) Contributions of four age groups to total CRC deaths attributable to all risk factors by SDIs and GBD regions in 1990 and 2019. (D) Contributions of different age groups in death cases attributable to risk factors in 1990 and 2019.

In 2019, there were 382,893 (95% UI, 332,745–435,105) attributable deaths in males and 248,857 (95% UI, 204,201–295,747) in females, which were 2.30 and 1.84 times of those in 1990, respectively (Table 1). Except for the low SDI region, the male to female ratios of ASDR increased year‐on‐year from 1990 to 2019, especially in high‐middle and middle SDI regions (Figure 3A). The increasing trend of gender gap in PAFs was more significant in low middle and low SD regions (Figure 3B).

Australasia experienced the largest average annual decline in ASDR in both sexes (AAPC = −1.808% in male, −1.685% in female). Eastern Europe, Western Europe, high‐income North America, and high‐income Asia Pacific with high SDI values showed a decreasing trend in ASDR. East Asia experienced the largest average annual increase in ASDR in males (AAPC = 2.189%), while this was the case for Western Sub‐Saharan Africa in females (AAPC = 1.087%). High‐income Asia Pacific experienced a significant decline in PAFs in males (change of PAFs = −5.06%), while this was the case for Eastern Europe in females (change of PAFs = −1.45%). Most of Latin America showed a decline in PAFs (Figure S1C,D). In most regions, the AAPC of ASDR and the change in PAFs were significantly higher in males than those in females.

In general, the attributable CRC death burden in males was significantly higher than that in females, and the current trends suggest that the gender gap will continue to widen in the future.

### 3.3 Age differences and trends in attributable CRC death burden

In order to better analyze the attributable CRC death trend, we divided every 5 years into one age group from 15 to 85 years plus, comprising 15 age groups.

In 2019, the deaths increased with age in higher (high and high middle) SDI regions, with the largest death occurring in the ≥85 years age group. In lower (middle, low middle, and low) SDI regions, the death peak occurred at a younger age, with the largest number in the 64–69, 70–74 year age groups. The proportion of premature deaths under 50 years of age was significantly higher in lower SDI regions. It is worth mentioning that, in higher SDI regions, the deaths in the age groups of 70 years and above increased significantly. In comparison, in lower SDI regions, deaths in the age groups of 30 years and above began to double or multiply compared to 1990 (Figure 3C).

In order to further analyze the regional gap in age, we simplified the age groups into four categories: 15–49 years, 50–69 years, 70–84 years, and ≥85 years of age (Figure 3D). The main CRC‐attributable death burden concentrated in the 70–84 age group in higher SDI regions but this was the 50–69 age group in lower SDI regions. From high to low SDI, the 15–49 age group accounted for 3%, 6%, 10%, 11%, and 15%, with an increase of 4.5%, 54.8%, 123.1%, 147.6%, and 163.3% compared to 1990, respectively. The ≥85 age group accounted for 26% and 15% in higher SDI regions, respectively, which were significantly higher than those in lower SDI regions. Among the 21 GBD regions, high‐income Asia‐Pacific region and Western Europe with high SDI values had the highest proportion of CRC‐attributable death burden in the ≥85 age group, accounting for 32% and 29%, respectively.

In contrast, Oceania and eastern sub‐Saharan Africa had the highest proportion of attributable CRC burden in the 15–49 age group, accounting for 18% and 17%, respectively.

From the above data, it is clear that the attributable CRC death burden tends to occur at a younger age in lower SDI regions.

### 3.4 Inadequate control of dietary and metabolic risk factors is the leading cause of CRC‐attributable deaths

**FIGURE 4:**
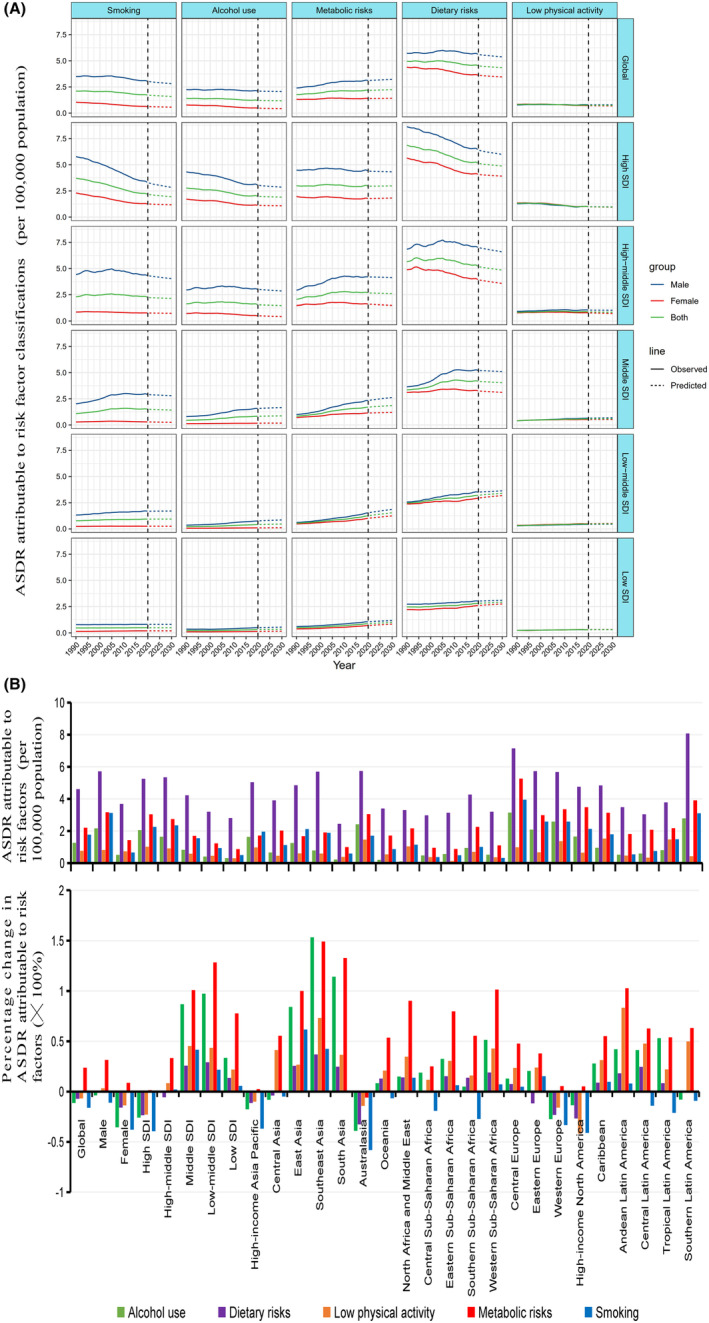
Trends and prediction of ASDR attributable to risk factor classifications in CRC by SDIs and GBD regions. (A) Prediction of ASDR attributable to risk factor classifications in CRC by SDIs. (B) ASDR in 2019 and its percentage change between 1990 and 2019 for CRC attributable to different risk factor classifications by SDIs and GBD regions.

Figure 4A presents the ASDRs attributable to risk factor classifications by SDIs from 1990 to 2019, and predicts the trends in the next 10 years. In global terms, the ASDRs attributable to dietary factors, metabolic factors, smoking, alcohol use, and low physical activity in 2019 were 4.61, 2.21, 1.77, 1.26, and 0.77 per 100,000 population, respectively. Over the past 30 years, ASDRs attributable to dietary risk, smoking, alcohol use, and low physical activity have declined by 7.1%, 16%, 11.3%, and 6.7%; in contrast, metabolic factors have increased by 23.7%. As a result, dietary factors remain the leading cause of colorectal‐attributable deaths. Metabolic factors play an increasingly significant role; they have jumped to second place and are expected to grow in prominence in the next decade.

**FIGURE 5:**
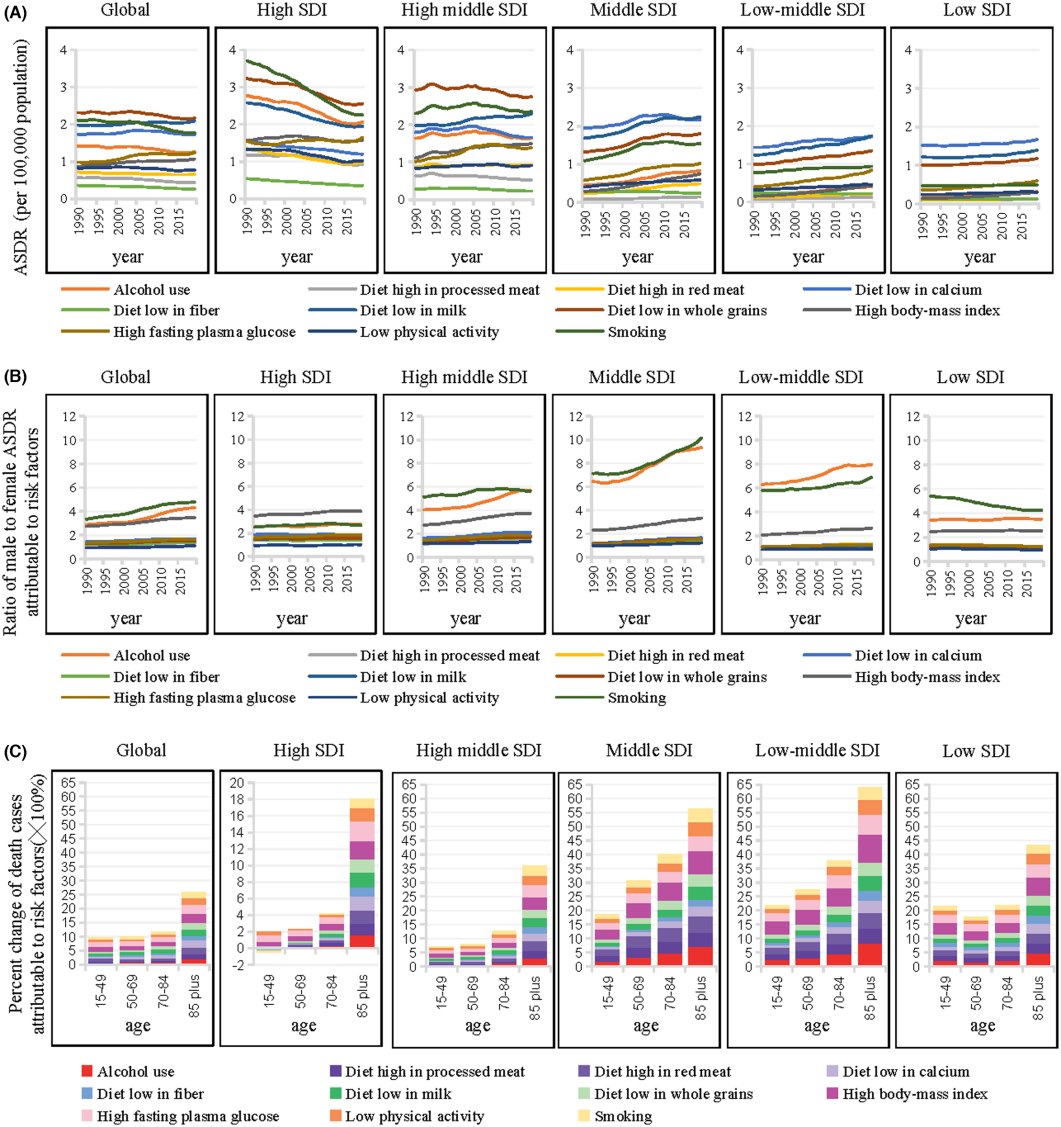
Trends in deaths and ASDR attributable to risk factors in CRC by SDI, sex and age groups. (A) Trends in ASDR attributable to risk factors in CRC by SDIs from 1990 to 2019. (B) Trends in male to female ratio for ASDR attributable to risk factors in CRC by SDIs from 1990 to 2019. (C) Percentage changes in CRC deaths attributable to risk factors by SDIs between 1990 and 2019 in four age groups.

The ASDRs attributable to dietary factors have decreased comprehensively in high SDI regions since 1990. Among the GBD regions, represented by Western Europe and Australasia, ASDRs have decreased significantly. At the national level, in most countries with high SDI values (≥0.812), the ASDR caused by dietary factors showed a downward trend. In high‐middle SDI regions, the ASDR decreased by 5.5%. Among the top four dietary risks for ASDRs, diet low in whole grains and calcium decreased by 6.19% and 7.69%, respectively, while diet low in milk increased by 15.94%. Among the GBD regions, the ASDRs caused by most dietary factors have decreased slightly in Eastern Europe while they were stable in Southern Latin America but increased in Central Europe. At the national level (SDI value ≥0.69), in Israel, Italy, Malta, and other countries, the ASDRs caused by dietary factors decreased. The lower SDI regions, including middle, low‐middle, and low SDI region, showed a consistent trend. Within 30 years, the ASDRs all increased significantly by 25.8%, 29.1%, and 13.5%. Among GBD regions, including Southeast Asia, South Asia, Latin America, and Africa, ASDRs caused by dietary factors showed an upward trend. The top three risk factors were diet low in milk, diet low in whole grains, and diet low in calcium. At the national level (SDI value ≤0.689), Cabo Verde, Equatorial Guinea, and Vietnam have the largest increases of ASDR attributable to dietary factors, by 3.93, 3.83, and 2.76 times compared to 1990, respectively (Figures 4B and 5A; Figures S2 and S3; Table S2). This indicates that poor control of dietary factors in the lower SDI regions contributes to the increased CRC‐attributable death burden.

The ASDRs attributable to metabolic factors showed a clear upward trend worldwide over the examined period. In high SDI regions, the ASDR slightly increased (5.47% by high fasting plasma glucose and 0.75% by high body‐mass index). Among the GBD regions, represented by high‐income Asia Pacific, high‐income North America, Western Europe, and Australasia, ASDRs were well controlled. At the national level (SDI value ≥0.812), the ASDRs were controlled smoothly, represented by Australia, Singapore, and Luxembourg. In high‐middle SDI regions, ASDRs significantly increased by 33.3% (34.09% by high fasting plasma glucose and 36.25% by high body‐mass index). Among the GBD regions, ASDRs all showed an upward trend to varying degrees, especially in Central Europe and Southern Latin America. At the national level (SDI value ≥0.69), most countries had an increasing trend in ASDRs. In lower SDI regions, the ASDRs doubled and the increase was more obvious by high body‐mass index than by high fasting plasma glucose. Among the GBD regions, the Latin America region (represented by Southern Latin America), North Africa and the Middle East, Southern Sub‐Saharan Africa, and Central Asia had larger ASDRs. In contrast, Southeast Asia, South Asia, Western Sub‐Saharan Africa, and Andean Latin America showed the most significant increases. At the national level (SDI value ≤0.689), the ASDRs in most countries and regions showed a relatively obvious growth trend (Figures 4B and 5A; Figures S2 and S3; Table S2). Based on these data, poor overall control of the metabolic factors contributes to the increased CRC‐attributable death burden.

The ASDRs attributable to smoking and alcohol use in CRC have decreased worldwide in past 30 years. In high SDI regions, ASDRs decreased by 39.27% and 25.88%, respectively, for these two factors, represented by high‐income Asia Pacific, high‐income North America, Western Europe, and Australasia. The ASDRs showed a stable trend in high‐middle regions. In lower (middle and low‐middle) SDI regions, represented by East Asia, Southeast Asia, North Africa and the Middle East, the ASDRs for smoking and alcohol use increased significantly by 41.64% and 86.88% in middle SDI regions, and 21.71% and 97.27%, respectively, in low‐middle SDI regions (Figures 4B and 5A; Figures S2 and S3; Table S2). Overall, in lower SDI regions, it is still necessary to increase the control of tobacco and alcohol.

The ASDR attributable to low physical activity was not high and showed an overall downward trend worldwide in the past 30 years. However, its burden trend distribution was extremely uneven; the high SDI regions, represented by high‐income Asia Pacific, high‐income North America, Western Europe, and Australasia, mainly showed a downward trend; whereas in Asia, Africa, and Latin America, there was even substantial growth in death burden caused by low physical activity (Figures 4B and 5A; Figures S2 and S3; Table S2). Therefore, in the lower SDI regions, low physical activity remains a concern.

From a global perspective, ASDRs attributable to smoking, alcohol use and high body‐mass index had the most obvious gender differences, with male to female ratios of 4.79, 4.30, and 3.47 times, respectively, in 2019. The gender difference caused by smoking and drinking was more significant in high middle, middle, low middle SDI regions and showed an obvious upward trend. In contrast, dietary risks and high body‐mass index were more pronounced in higher SDI regions (Figure 5B; Figure S4).

In high SDI regions, in the 15–49 age group, the increase in deaths attributable to smoking and alcohol use was negative and that by dietary factors was close to zero, which may explain the obvious decline trend of premature death in the high SDI region within 30 years. In higher SDI regions, the increase in deaths attributable to all factors concentrated in the ≥85 age group. In contrast, in lower SDI regions, increases were also evident in the 15–49, 50–69, and 70–84 age groups. Different increases in these risk factors across age groups led to a higher burden in younger age groups in lower SDI regions. Notably, in each SDI region, the increase in deaths attributable to metabolic factors was not significantly lower in the 15–49 age group than in other groups. This trend was distinct from other factors, suggesting that metabolic factors play a key role in the global burden of premature deaths (Figure 5C; Figure S5).

## 4 DISCUSSION

The burden of CRC is enormous worldwide, ranked third in morbidity and second in mortality among all malignant tumors.^3^, ^20^ At the same time, most CRC deaths are attributable to modifiable risk factors.^8^, ^9^ In 2019, the population‐attributable fraction attributable to CRC accounted for 58%. Therefore, the prevention and control of modifiable risk factors based on epidemic characteristics, precision and local conditions can significantly help control the CRC burden. Unlike other efforts focused on analyzing the morbidity and mortality rates, our study provides an in‐depth analysis of trends in CRC mortality attributable to risk factors by region, country, gender, age group, and timeline based on the GBD 2019 data. Overall, the attributable CRC deaths increased steadily from 301,767 in 1990 to 631,750 in 2019, with a slight downward trend in ASDRs, which suggested that population growth and aging might be responsible for the increase in absolute deaths. Notably, dietary factors remain major drivers of CRC worldwide.^27^ Metabolic factors are poorly controlled globally and have jumped to second place. Due to the lack of dual control of dietary and metabolic factors, CRC‐related deaths have shifted from higher to lower SDI regions.

Numerous studies have indicated that the burden of CRC can be seen as a marker of socioeconomic development.^28^, ^29^ In higher SDI countries, such as Austria, the United States, and Australia, in recent decades, CRC‐related deaths caused by various factors have been reduced after strengthening the relevant health promotion measures, such as the implementation of smoking and alcohol restriction policies, increasing fiber intake, encouraging exercise, and actively controlling metabolic‐related diseases.^30^ The practice and implementation of CRC cancer treatment and management approaches in these countries, such as stool immunochemical testing, CT colonography, colorectal screening, and early lesion resection, have also been beneficial in reducing CRC mortality rates.^16^, ^31^ The United States began insurance coverage for CRC screening as early as 1998,^32^ and their professional Multi‐Society Task Force (MSTF) of CRC develops and regularly updates CRC screening plans.^33^, ^34^, ^35^ Australia launched the National Bowel Screening Program (NBCSP) in 2006, followed by its implementation and gradual improvement.^36^ In contrast, in lower SDI regions and some transition countries represented by China, Mexico and India, there is a marked increase in CRC morbidity and mortality because of great changes in lifestyle and diet, increased smoking and metabolic diseases, which are caused by rapid economic transformation, industrialization, urbanization, and globalization. At the same time, inadequate national screening prevention policies and poorly designed healthcare improvements are also contributing factors.^28^, ^37^, ^38^

The attributable death burden in CRC is significantly higher in males than females in terms of number and ASRs and shows significant regional heterogeneity. The gender gap is more marked in the high middle SDI and middle SDI regions, which is increasing year‐on‐year and expected to further expand. Differences in behavioral factors account for the gender gap in the CRC‐attributable death burden, such as smoking, alcohol consumption, stress, lifestyle, and so on.^39^, ^40^ This is consistent with our findings that ASDRs attributable to smoking, alcohol and metabolic factors are significantly higher in males than females, especially in areas with relatively low SDI values, such as middle SDI and low middle SDI regions.^41^, ^42^ Therefore, a greater awareness of risk factors and better management is crucial to narrowing the gender gap.

Our research highlights that the proportion of deaths at 70 years of age and older has significantly risen worldwide since 1990, which is closely related to the progress of global medical care and the advancement of population aging.^43^, ^44^ The peak onset age is significantly younger in lower SDI than in higher SDI regions. However, it is worth noting that in lower SDI regions, represented by Africa, the cases of premature onset and premature death (<50 years old) accounting for a relatively high proportion has increased significantly. Studies have suggested that early onset is associated with a Western‐style diet, obesity, physical inactivity, and antibiotic use, especially during prenatal to adolescence.^45^ In higher SDI regions, promoting colonoscopy and early cancer screening may be partially responsible for this trend in premature onset. Therefore, in lower SDI regions, it is necessary to increase popular science‐based healthy diets and lifestyles and strengthen the management and early screening of young people in high‐risk groups, such as those with a family history of smoking, alcohol use, and so on. At the same time, it is important to actively promote appropriate early screening measures for colorectal cancer, including early fecal screening, colonoscopy promotion, among other measures.^46^, ^47^

Various risk factors underlie the current colorectal cancer death burden. Therefore, implementing multiple risk factor interventions according to local conditions is a key measure to prevent and control colorectal cancer.^48^ It is also worth noting that dietary factors and metabolic factors remain the leading causes of uncontrolled CRC‐related deaths, and the management of these two factors is still the focus of CRC prevention and control. In lower SDI regions, irrational diet and lifestyle, restrictions on primary care preventive medical resources and economic development, and limited ability to control metabolic factors are mainly responsible for the increased deaths caused by metabolic factors. As reported previously, high BMI and high blood glucose in CRC have increased dramatically over the past few decades, resulting in a massive burden of premature deaths.^49^, ^50^, ^51^, ^52^, ^53^ Lower SDI regions need to urgently implement long‐term layout according to national conditions to reverse this unfortunate trend, such as encouraging outdoor exercise, losing weight, strengthening blood sugar management, and educating healthy dietary structure, especially the intake of grains, calcium, and milk.

CRC‐related deaths caused by smoking and alcohol use have decreased significantly because of the implementation of health promotion and policies to combat tobacco and alcohol worldwide, especially in higher SDI regions.^54^, ^55^ However, particular attention should be paid to lower SDI regions, such as East and Southeast Asia, North Africa, the Middle East, and western Sub‐Saharan Africa, where the burden of CRC caused by smoking and alcohol use has shown a clear upward trend. Furthermore, smoking and alcohol use also lead to gender differences in CRC‐related ASDR. Therefore, it is an essential part of CRC prevention and control to strengthen the implementation of tobacco control and alcohol restriction policies, with particular emphasis on lower SDI regions.

The effective control of risk factors holds great promise in reducing CRC‐related mortality. In recent years, higher SDI regions have made great achievements in relevant prevention and control measures, while lower SDI regions are lagging behind due to economic and social development constraints. These regions and countries must urgently focus on solving the key risk factors according to their economic and medical conditions. More effective prevention and control policy formulation are necessary to facilitate the control of CRC burden.

Based on the GBD database, our study is the first to comprehensively and systematically analyze the attributable risk of CRC death burden and its temporal trends, as well as to predict future trends in this burden attributable to different risk factors. Meanwhile, there are some inevitable limitations to our study. First, the data sources available for GBD have some inherent restraints, such as the absence of population‐based cancer registries in some countries and the incomplete management of cancer registry data in many low‐SDI regions and countries. Second, the availability of data to estimate risk factor exposure is sometimes low, with many data sources not providing sufficient information to assess potential measurement errors or biases. Where information is available, GBD models have used methods to correct for systematic bias in the risk exposure data, while residual measurement bias may persist and this may vary over time. Finally, the risk factors for colorectal cancer included in this study are based on our current understanding of colorectal cancer risk factors. As the available knowledge expands, there may be further important risk factors that need to be included in future iterations of GBD studies, among others.

## 5 CONCLUSION

In summary, our analysis of the attributable CRC death burden over the period of 2009–2019 showed that it gradually shifted from higher SDI to lower SDI regions. The transformation was mainly due to the inadequate control of dietary and metabolic risk factors, especially in lower SDI regions. The ASDR of males was significantly higher than that of females, and the gap is expected to further expand. This gender difference is mainly due to the differential distribution of risk factors such as smoking, alcohol use and metabolic factors. The high premature attributable CRC death risk in lower SDI regions require the implementation of national‐level strategies to reverse this situation.

## AUTHOR CONTRIBUTIONS

**Ning Zhu:** Conceptualization (equal); data curation (equal); formal analysis (equal); methodology (equal); visualization (equal); writing – original draft (equal); writing – review and editing (equal). **Yan Zhang:** Conceptualization (equal); formal analysis (equal); methodology (equal); software (lead); visualization (lead); writing – original draft (equal); writing – review and editing (equal). **Mi Mi:** Data curation (equal); formal analysis (equal); methodology (equal); visualization (equal); writing – original draft (equal); writing – review and editing (equal). **Yuwei Ding:** Data curation (supporting); formal analysis (supporting); methodology (supporting); visualization (supporting). **Shanshan Weng:** Data curation (supporting); methodology (supporting); resources (supporting). **Jia Zheng:** Formal analysis (supporting); methodology (supporting). **Yang Tian:** Data curation (supporting); formal analysis (supporting); methodology (supporting). **Ying Yuan:** Conceptualization (equal); project administration (lead); supervision (lead); validation (lead); writing – review and editing (equal).

## FUNDING INFORMATION

This study was supported by the Key R&D Program of Zhejiang Province (2021C03125 to Ying Yuan), the National Natural Science Foundation of China (81872481 to Ying Yuan, 82000619 to Yang Tian) and Leading Innovative and Entrepreneur Team Introduction Program of Zhejiang (2019R01007 to Ying Yuan).

## CONFLICT OF INTEREST STATEMENT

The authors declare that the research was conducted in the absence of any commercial or financial relationships that could be construed as a potential conflict of interest.

## Supporting information


Figure S1.

Table S1.

Table S2.

## Data Availability

The datasets supporting the conclusions of this article are included within the article.
