# Supplementary material for: The death burden of colorectal cancer attributable to modifiable risk factors, trend analysis from 1990 to 2019 and future predictions
Source: Cancer Med. 2024 Mar 28;13(7):e7136. doi: 10.1002/cam4.7136 (PMC10973881; doi:10.1002/cam4.7136)

**Figure S1. Sex differences of ASDR and PAFs attributable to all risk factors in CRC by different SDIs and GBD regions.** (A) The ASDR in males and females by SDIs and GBD regions in 1990 and 2019. (B) The PAFs in males and females by SDIs and GBD regions in 1990 and 2019. (C) The AAPCs of ASDR in males and females by SDIs and GBD regions from 1990 to 2019. (D) The changes of PAF in males and females by SDIs and GBD regions from 1990 to 2019.


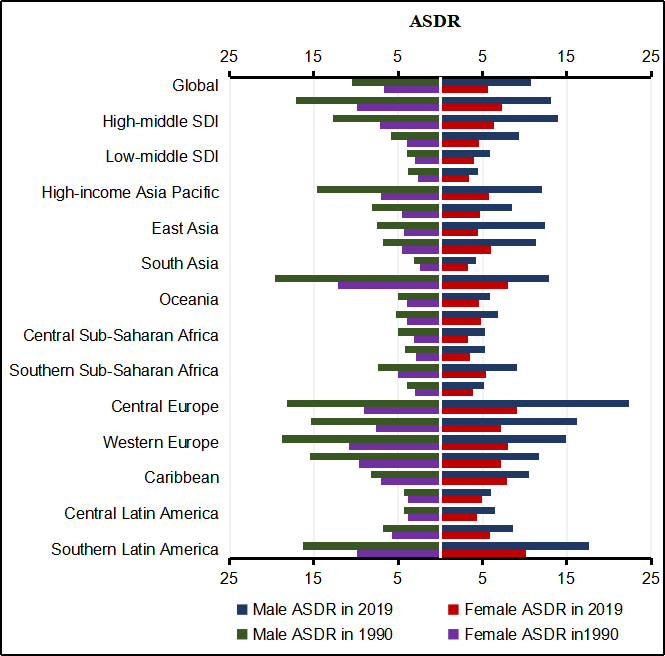

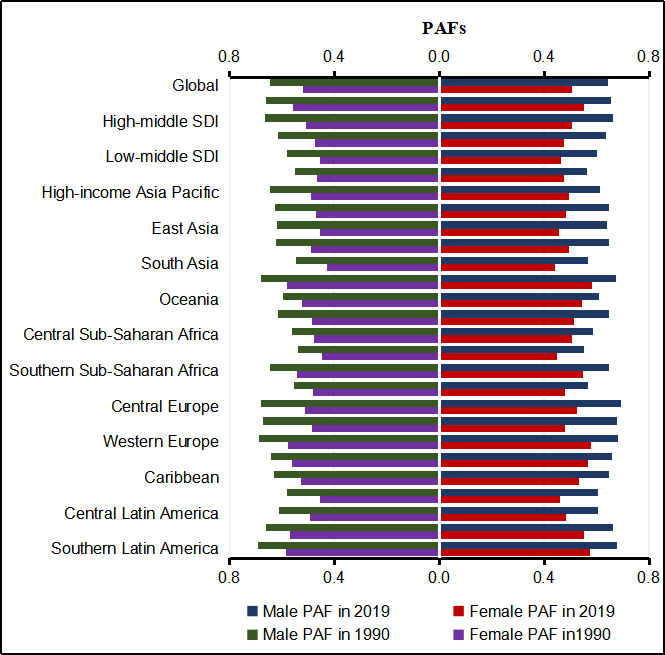


A

B


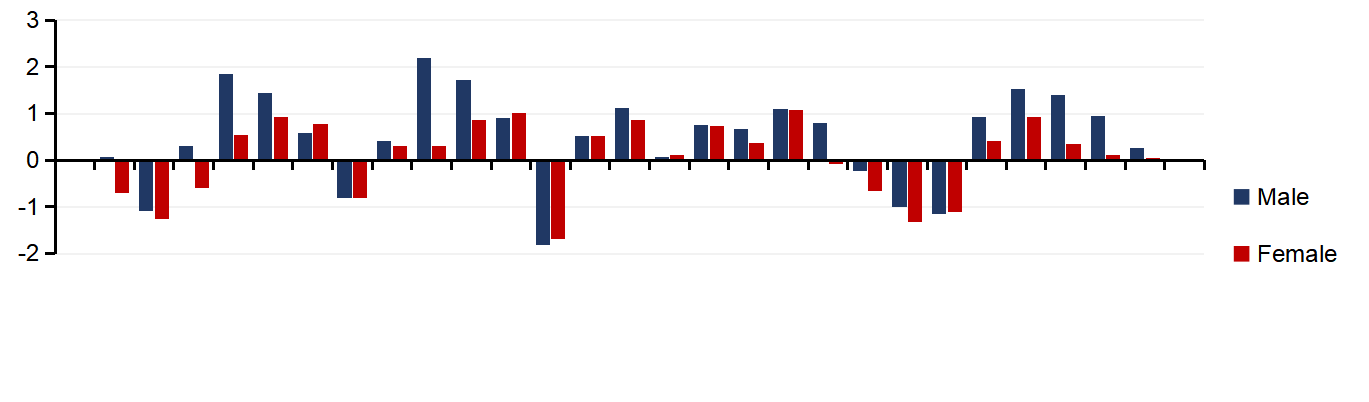


C


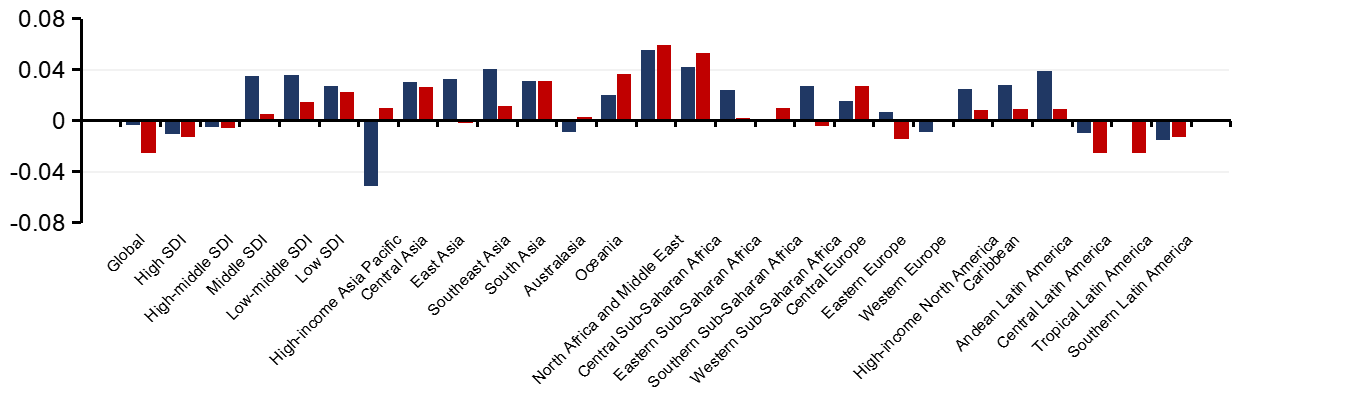


D

AAPC of ASDR attributable to risk factors ( 100%)

Percentage change in PAFs attributable to risk factors (* 100%)

**Figure S2. Comparison of the rankings of ASDR attributable to risk factors in CRC by SDIs between 1990 and 2019.**


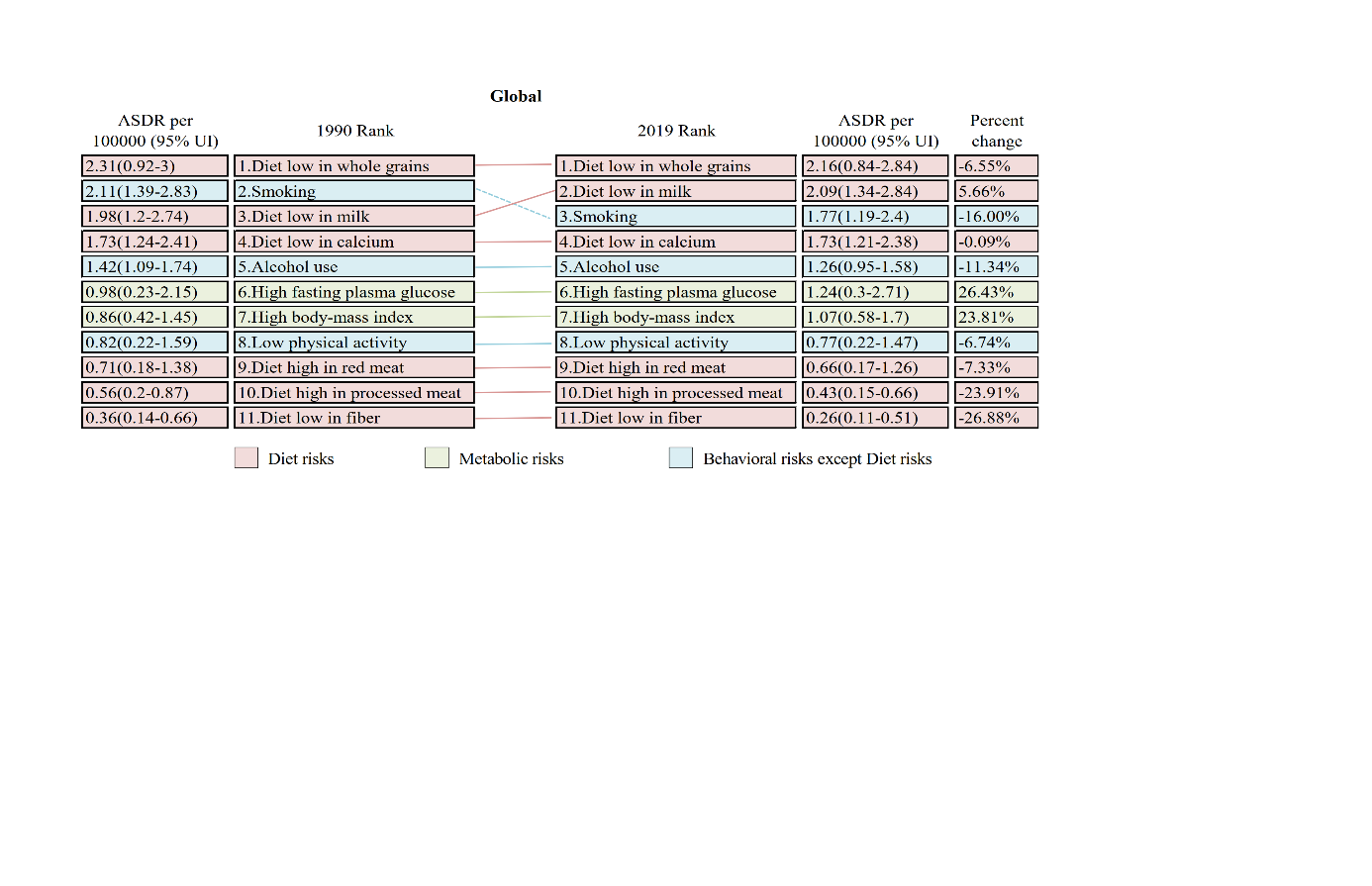

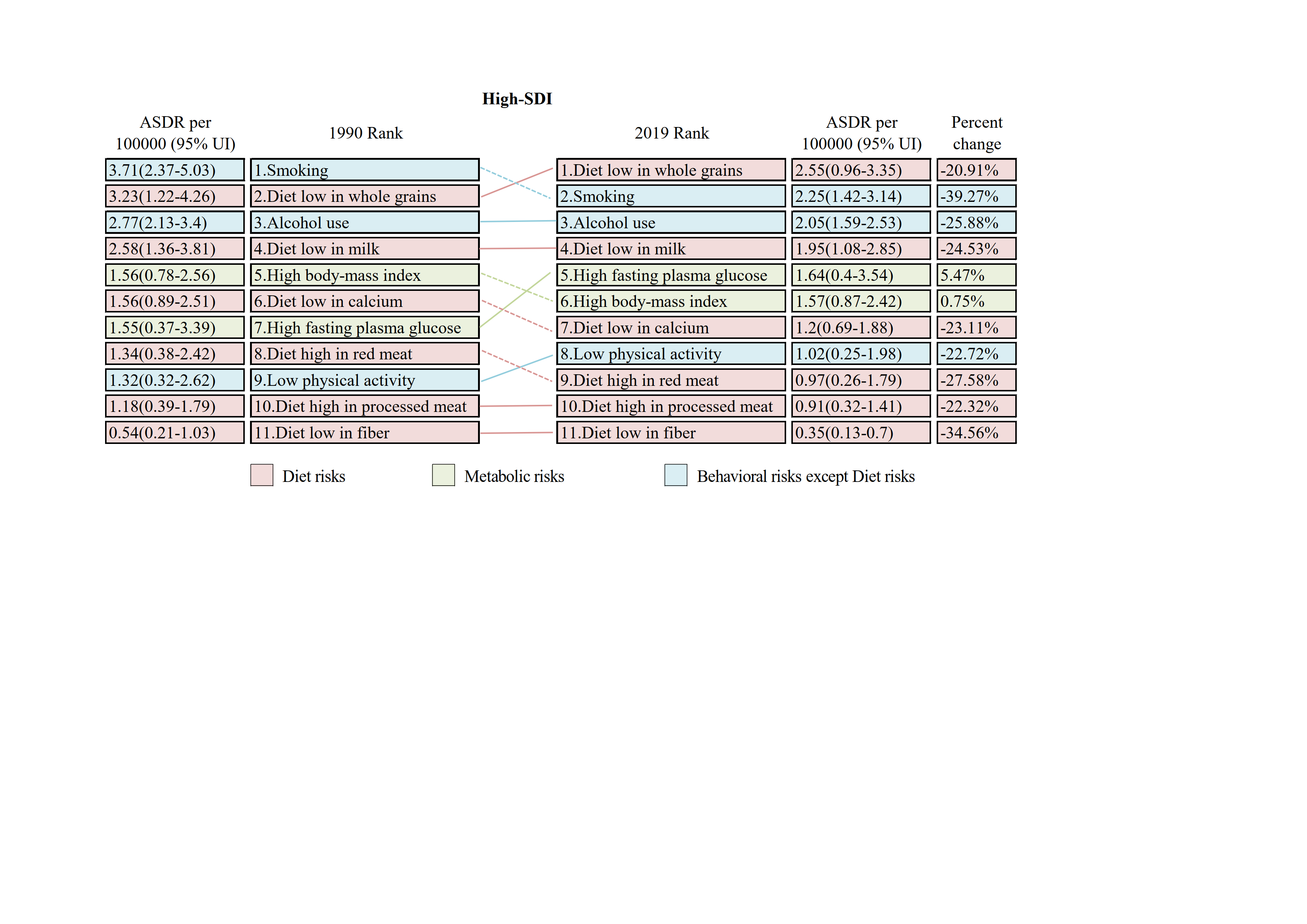

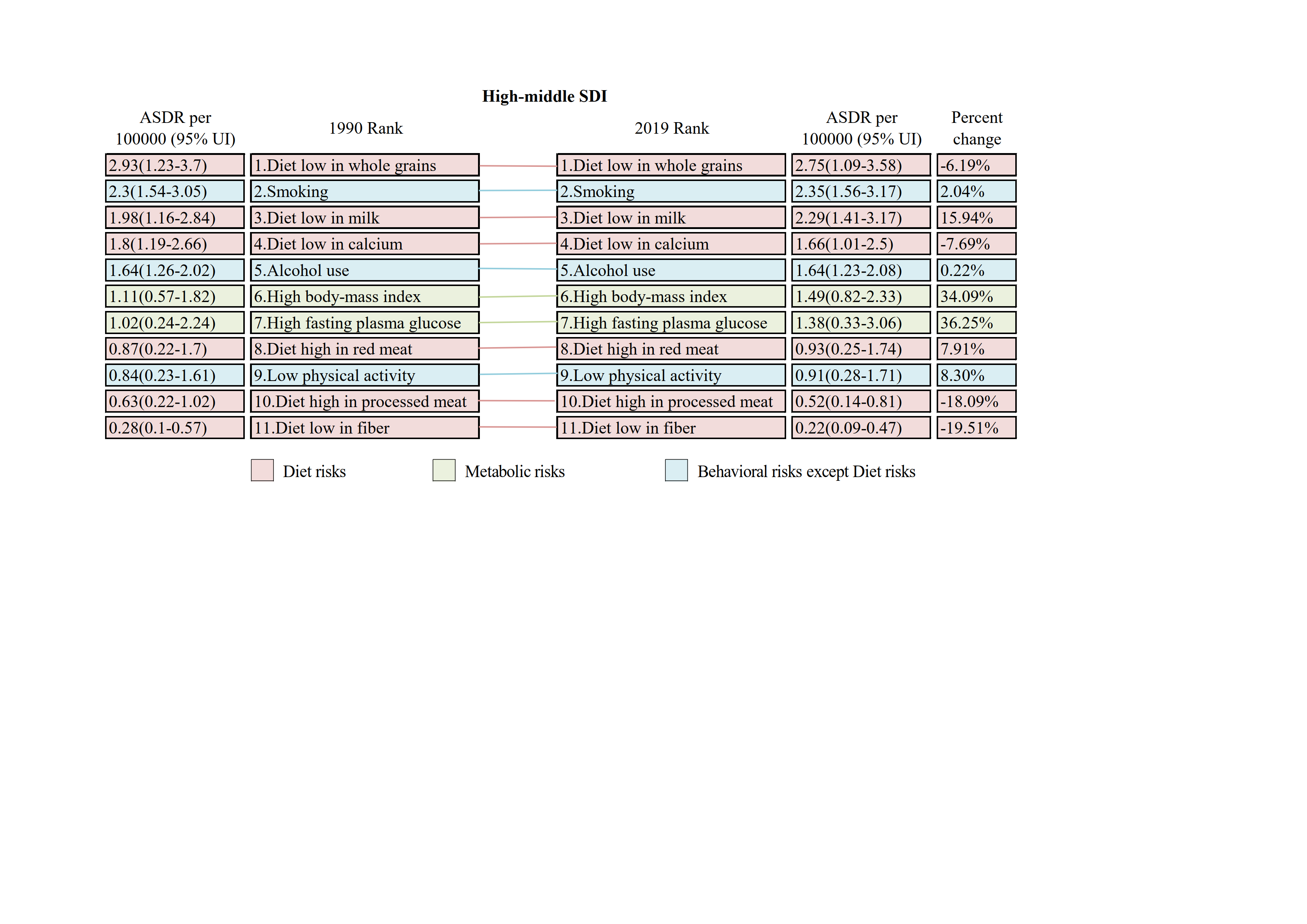

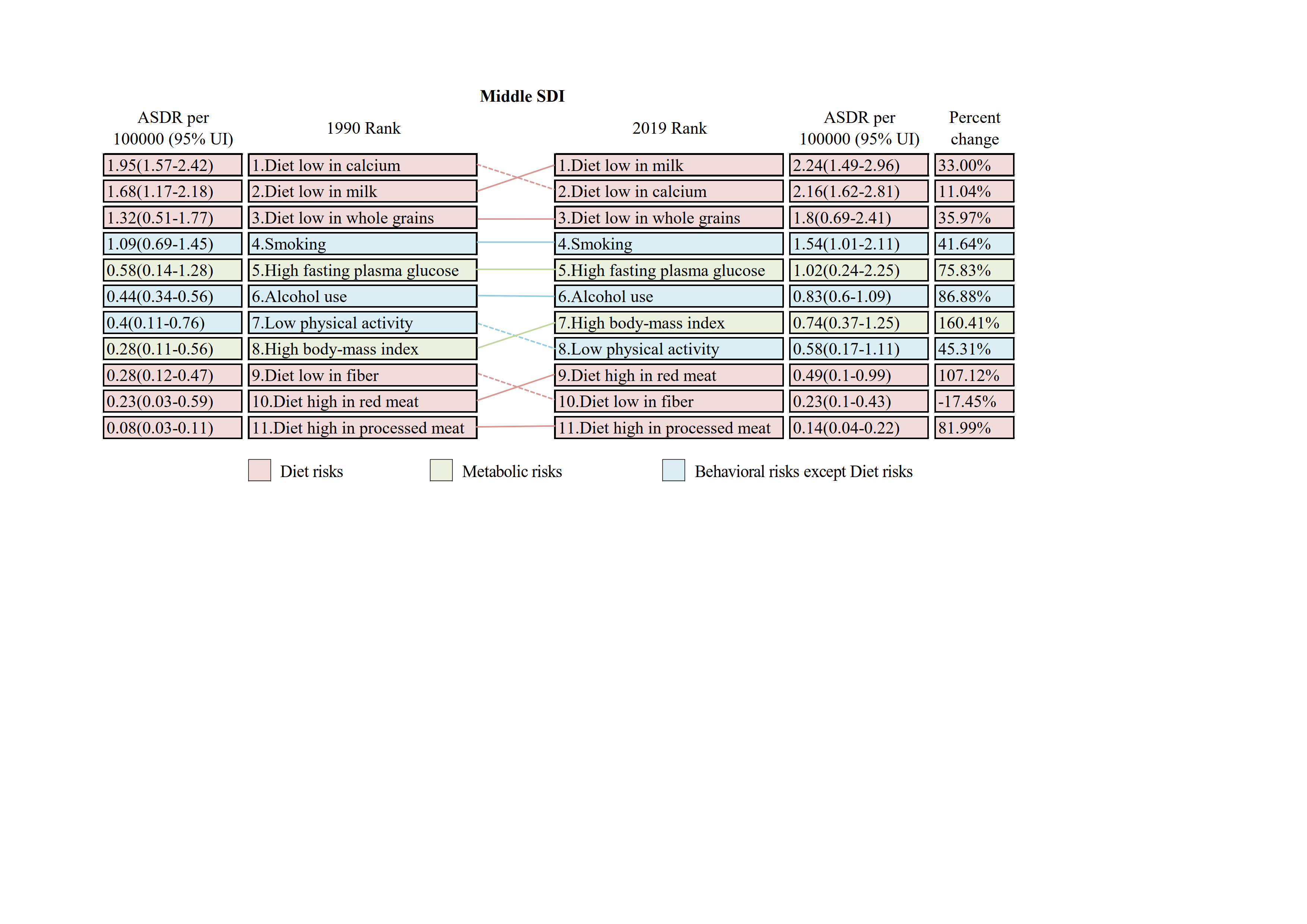

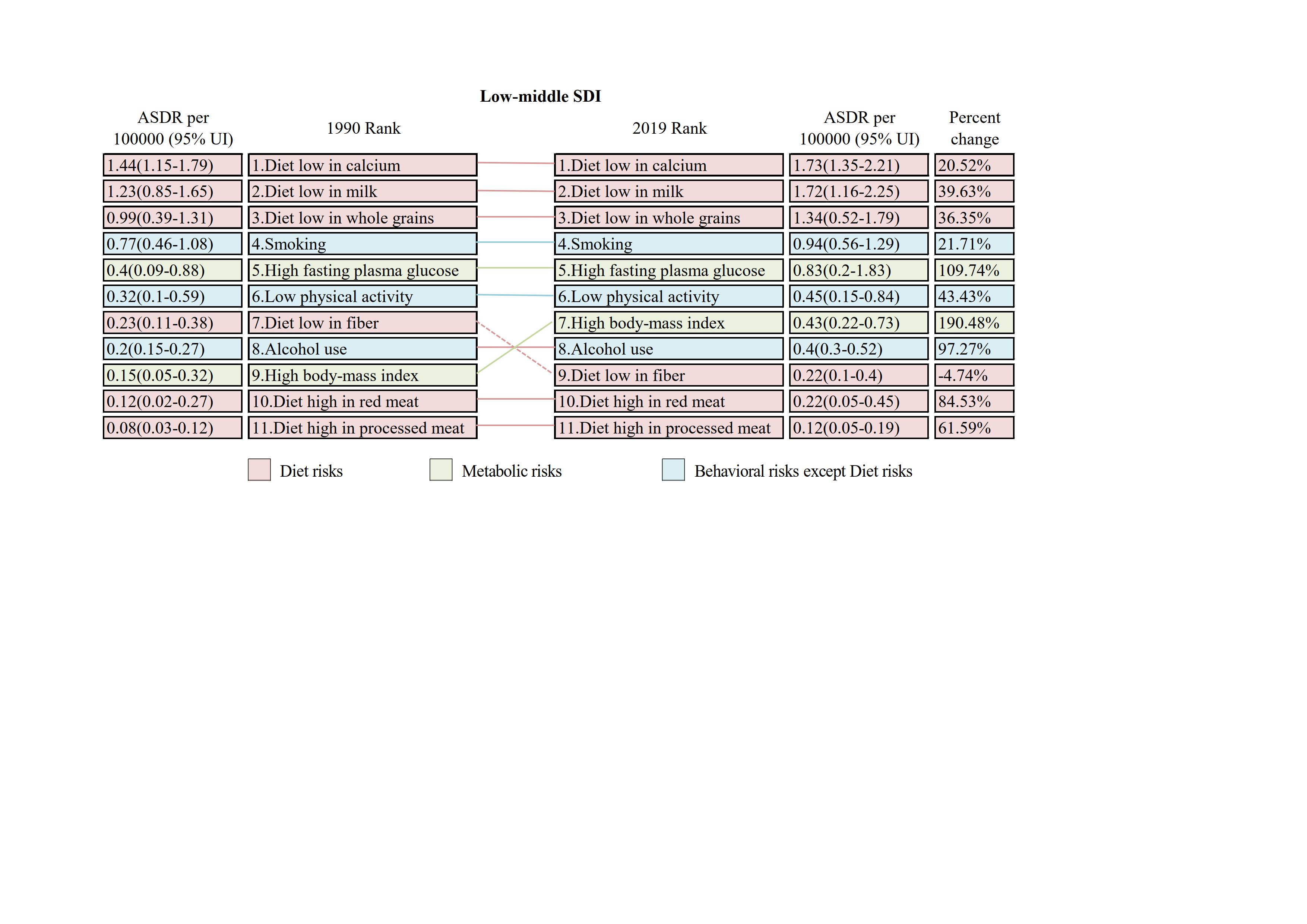

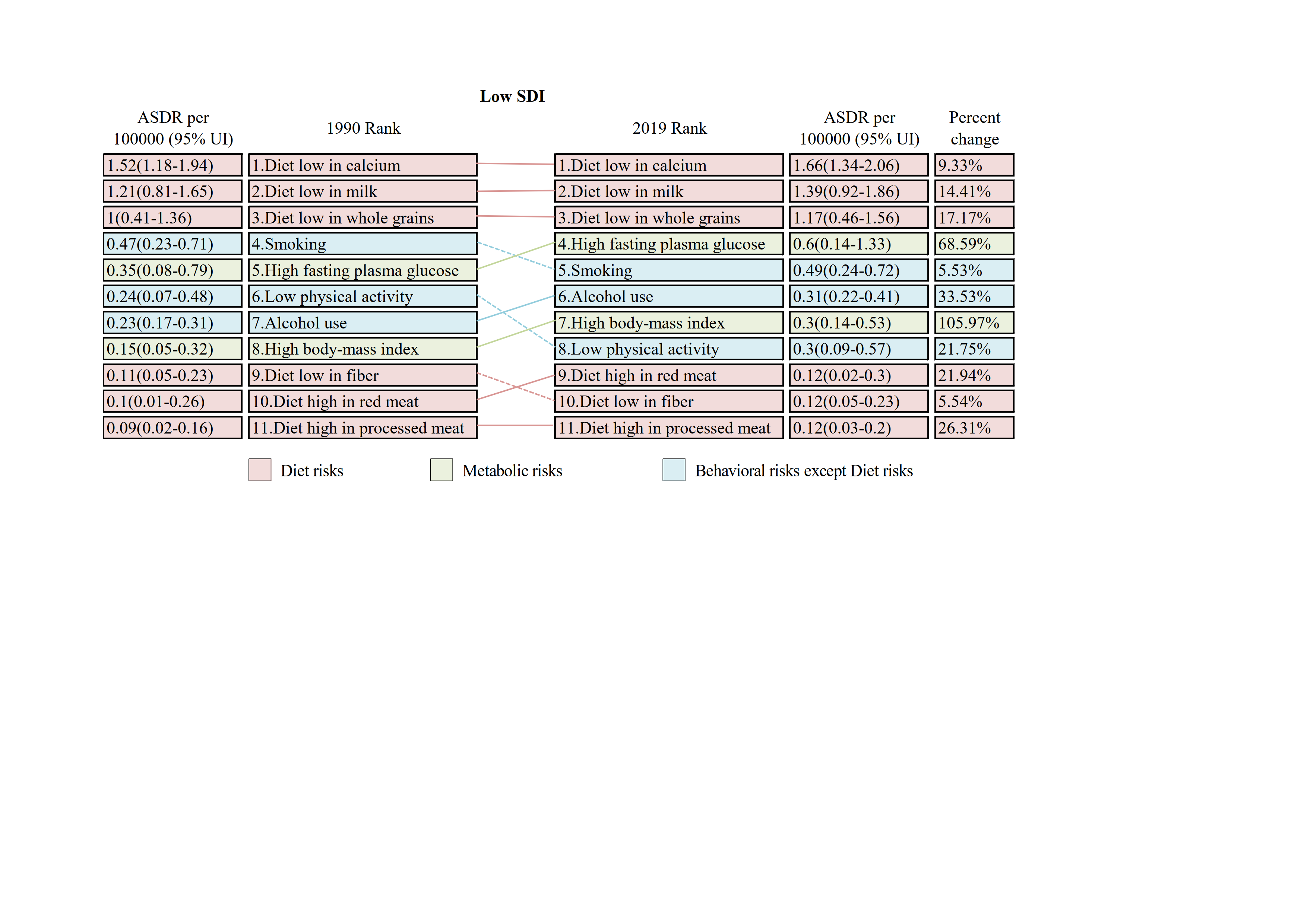


A

D

B

C

E

F

**Figure S3. The trends of ASDR attributable to risk factors in CRC by GBD regions from 1990 to 2019.**


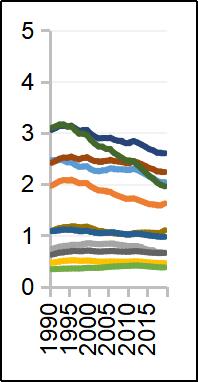


High-income Asia Pacific

ASDR (per 100,000 population)


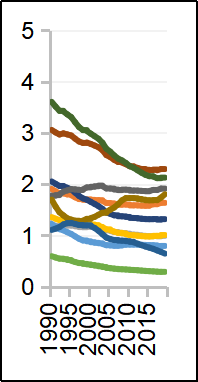


High-income North America


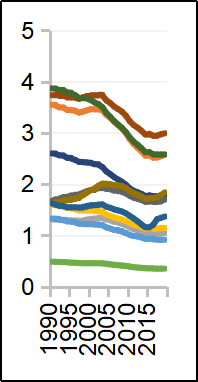


Western Europe


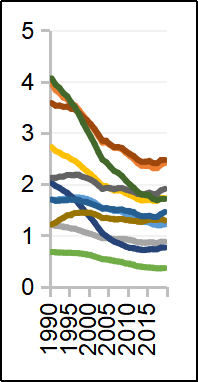


Australasia


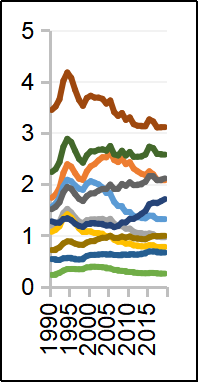


Eastern Europe


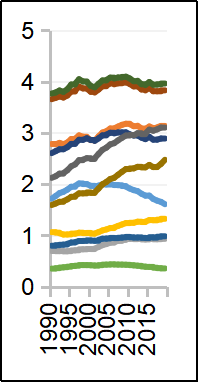


Central Europe


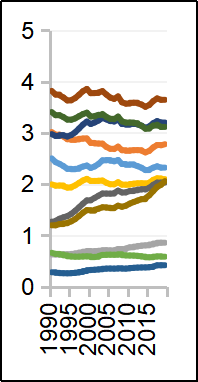


Southern Latin America


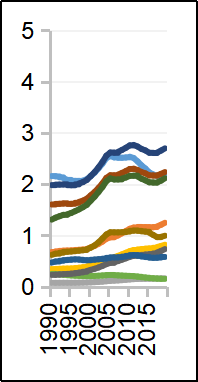


East Asia


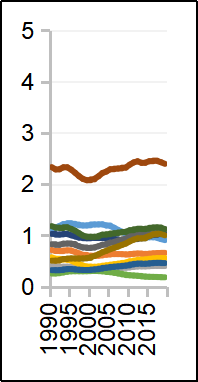


Central Asia


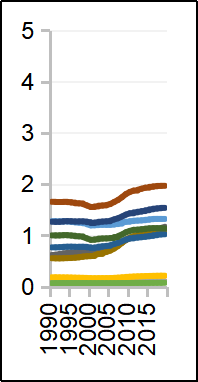


North Africa and Middle East


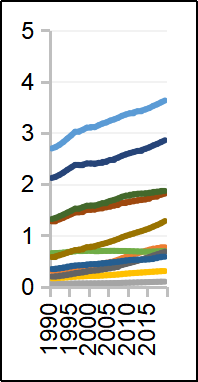


Southeast Asia


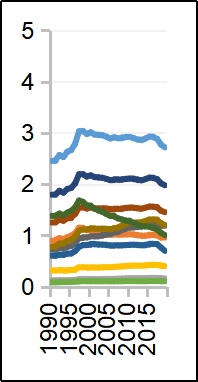


Southern Sub-Saharan Africa


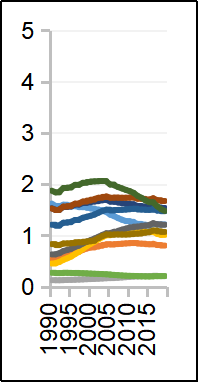


Tropical Latin America


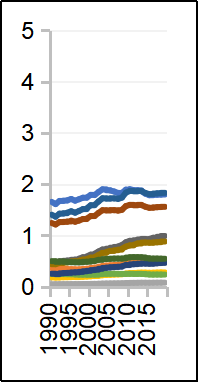


Andean Latin America

ASDR (per 100,000 population)

SDI=0.873

SDI=0.860

SDI=0.843

SDI=0.840

SDI=0.793

SDI=0.788

SDI=0.721

SDI=0.691

SDI=0.660

SDI=0.660

SDI=0.644

SDI=0.642

SDI=0.640

SDI=0.632

ASDR (per 100,000 population)


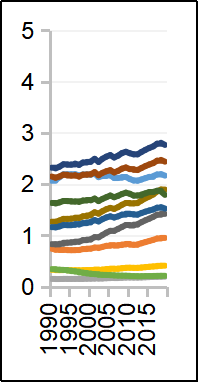


Caribbean


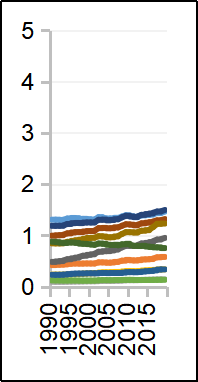


Central Latin America


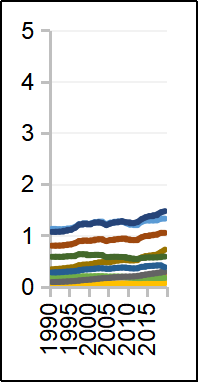


South Asia


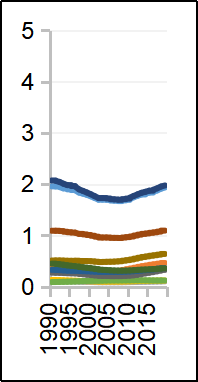


Central Sub-Saharan Africa


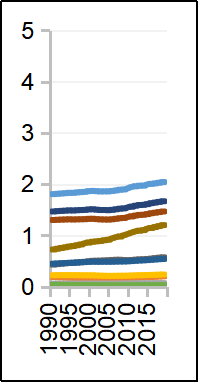


Oceania


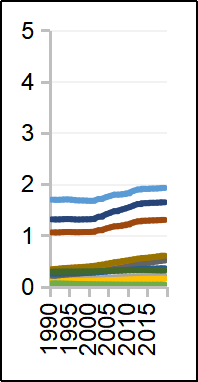


Western Sub-Saharan Africa


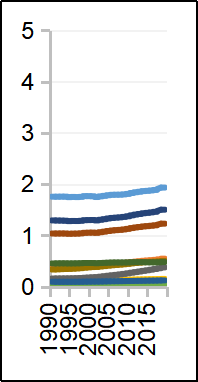


Eastern Sub-Saharan Africa


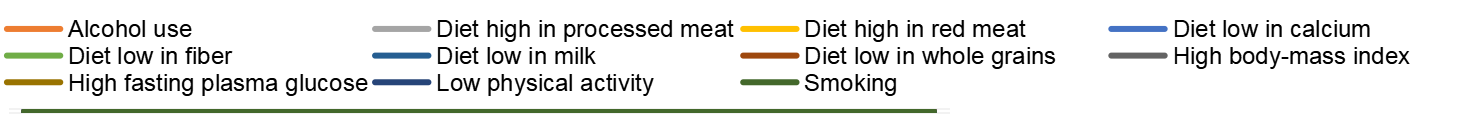


year

year

year

year

year

year

year

SDI=0.631

SDI=0.626

SDI=0.543

SDI=0.470

SDI=0.452

SDI=0.448

SDI=0.405

**Figure S4. Male to female ratios of ASDR attributable to risk factors in CRC by GBD regions from 1990 to 2019.**

East Asia

Central Asia

North Africa and Middle East

Southeast Asia

Southern Sub-Saharan Africa

Tropical Latin America

Andean Latin America

Ratio of male to female ASDR attributable to risk factors


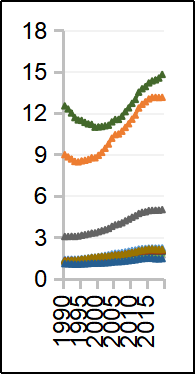

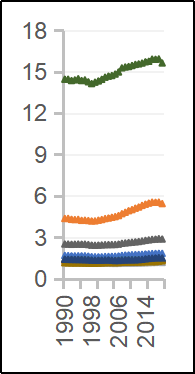

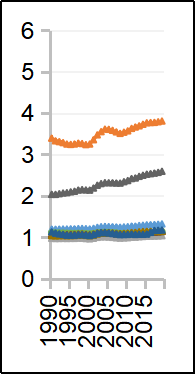

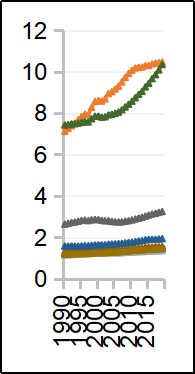

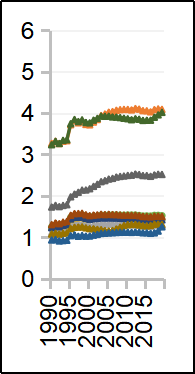

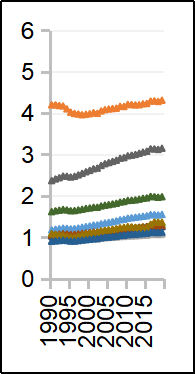

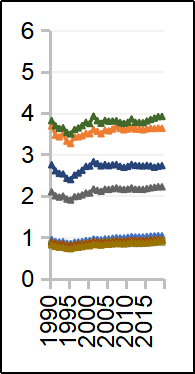


Ratio of male to female ASDR attributable to risk factors

Caribbean

Central Latin America

South Asia

Central Sub-Saharan Africa

Oceania

Western Sub-Saharan Africa

Eastern Sub-Saharan Africa


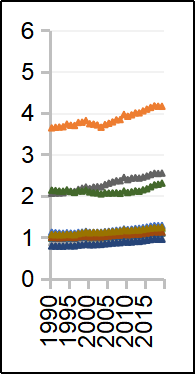

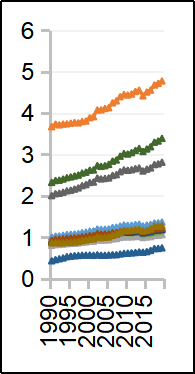

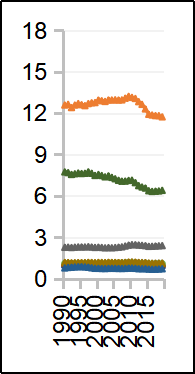

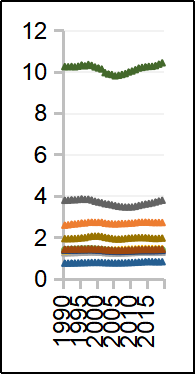

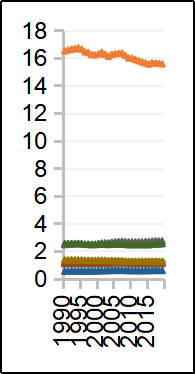

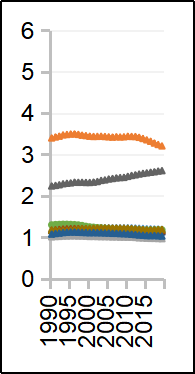

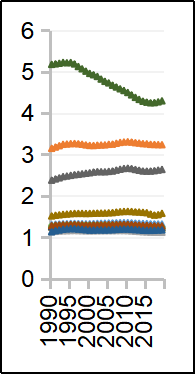

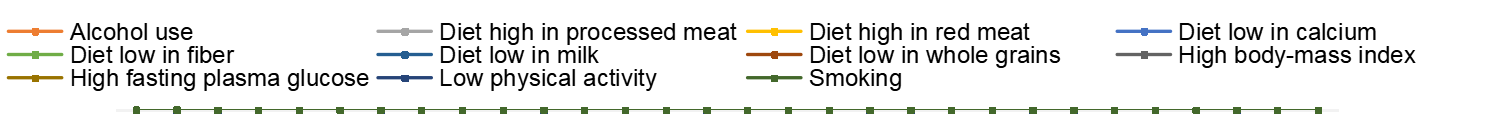


year

year

year

year

year

year

year

SDI=0.691

SDI=0.660

SDI=0.660

SDI=0.644

SDI=0.642

SDI=0.640

SDI=0.632

SDI=0.631

SDI=0.626

SDI=0.543

SDI=0.470

SDI=0.452

SDI=0.448

SDI=0.405

Ratio of male to female ASDR attributable to risk factors

High-income Asia Pacific

High-income North America

Western Europe

Australasia

Eastern Europe

Central Europe

Southern Latin America


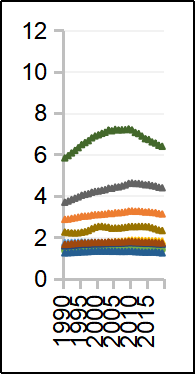

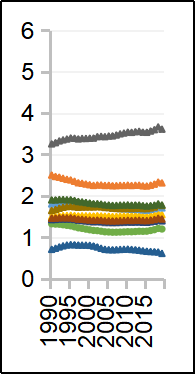

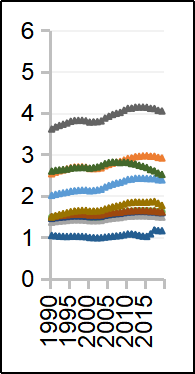

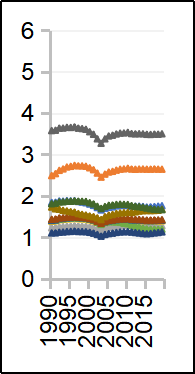

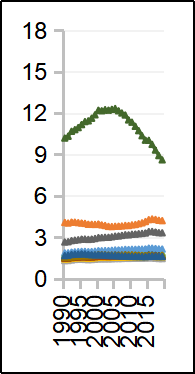

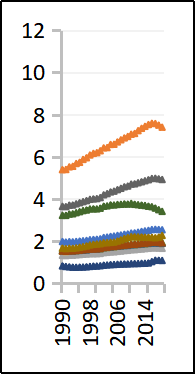

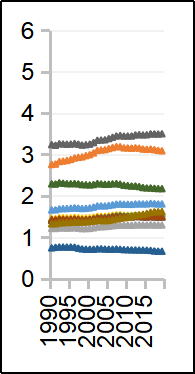


SDI=0.873

SDI=0.860

SDI=0.843

SDI=0.840

SDI=0.793

SDI=0.788

SDI=0.721

**Figure S5. The percent changes of age groups in deaths attributable to risk factor classifications in CRC by SDIs between 1990 and 2019.**

Percent change of death cases attributable to risk factors(*100%)

Global

High SDI

High middle SDI

Middle SDI

Low-middle SDI

Low SDI


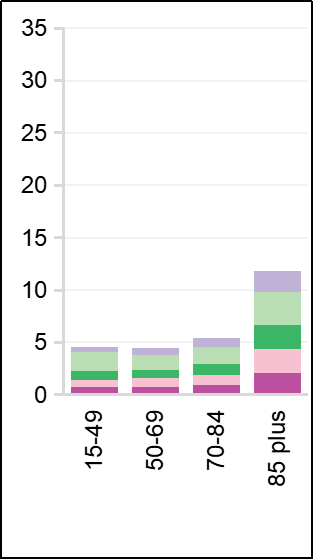

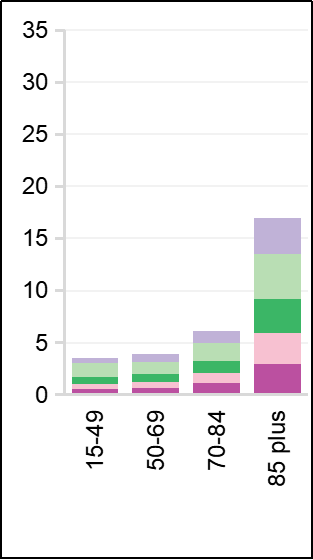

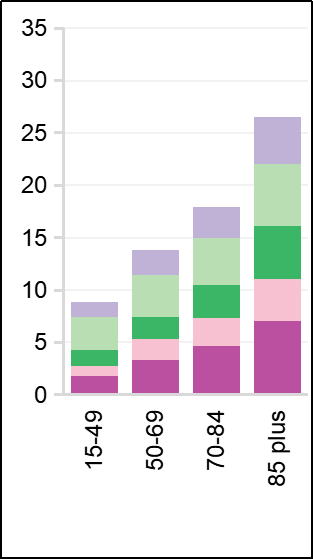

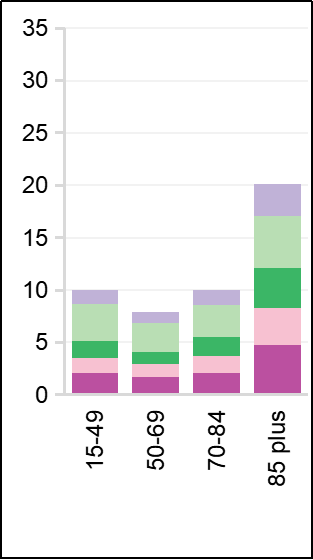


age

age

age

age

age

age


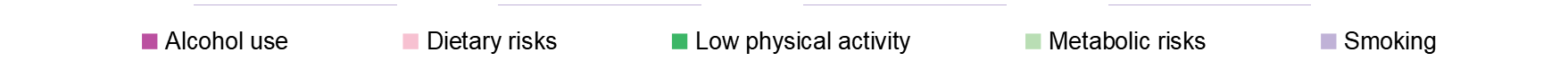

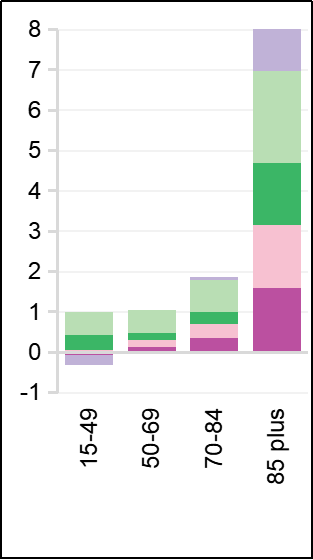

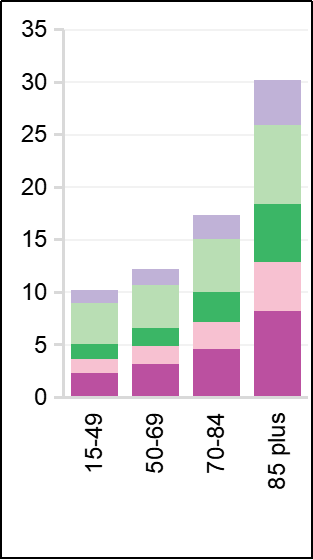

Supplement: Supplementary file 1 — Figure S1. [file CAM4-13-e7136-s003.docx]
